# The Long Read Transcriptome of Rice (*Oryza sativa* ssp. *japonica* var. Nipponbare) Reveals Novel Transcripts

**DOI:** 10.1186/s12284-022-00577-1

**Published:** 2022-06-11

**Authors:** Sharmin Hasan, Lichun Huang, Qiaoquan Liu, Virginie Perlo, Angela O’Keeffe, Gabriel Rodrigues Alves Margarido, Agnelo Furtado, Robert J. Henry

**Affiliations:** 1 Queensland Alliance for Agriculture and Food Innovation, University of Queensland, Brisbane, 4072 Australia; 2 Department of Botany, Jagannath University, Dhaka, 1100 Bangladesh; 3 College of Agriculture, Yangzhou University, Jiangsu, 225009 China; 4 Departamento de Genética, Escola Superior de Agricultura “Luiz de Queiroz”, Universidade de São Paulo, Piracicaba, São Paulo 13418-900 Brazil; 5 ARC Centre of Excellence for Plant Success in Nature and Agriculture, University of Queensland, Brisbane, 4072 Australia

**Keywords:** Rice transcriptome, Iso-sequencing, Full-length transcripts, Alternative splicing isoforms, Novel isoforms, Splicing junctions

## Abstract

**Background:**

High-throughput next-generation sequencing technologies offer a powerful approach to characterizing the transcriptomes of plants. Long read sequencing has been shown to support the discovery of novel isoforms of transcripts. This approach enables the generation of full-length sequences revealing splice variants that may be important in regulating gene action. Investigation of the diversity of transcripts in the rice transcriptome including splice variants was conducted using PacBio long-read sequence data to improve the annotation of the rice genome.

**Results:**

A cDNA library was prepared from RNA extracted from leaves, roots, seeds, inflorescences, and panicles of *O. sativa* ssp. *japonica* var Nipponbare and sequenced on a PacBio Sequel platform. This produced 346,190 non-redundant full-length non-chimeric reads (FLNC) resulting in 33,504 high-quality transcripts. Half of the transcripts were multi-exonic and entirely matched with the reference transcripts. However, 14,874 novel isoforms were also identified resulting predominantly from intron retention and at least one novel splice site. Intron retention was the prevalent alternative splicing event and exon skipping was the least observed. Of 73,659 splice junctions, 12,755 (17%) represented novel splice junctions with canonical and non-canonical intron boundaries. The complexity of the transcriptome was examined in detail for 19 starch synthesis-related genes, defining 276 spliced isoforms of which 94 splice variants were novel.

**Conclusion:**

The data reveal the great complexity of the rice transcriptome. The novel transcripts provide new insights that may be a key input in future research to improve the annotation of the rice genome.

**Supplementary Information:**

The online version contains supplementary material available at 10.1186/s12284-022-00577-1.

## Background

High-quality genome and transcriptome sequences are fundamental to the understanding of the structural and functional genomics of plants. The transcriptome characterizes a complete set of transcribed regions throughout the genome. The analysis of the transcriptome provides insights into gene structure, gene expression, and regulation including the abundance of a transcript, alternative splicing isoforms, or protein isoforms, and the developmental or tissue-specific pattern of gene expression of a trait or allele-specific expression (Manzoni et al. 2018). This information is crucial for a better understanding of cellular and tissue metabolism dynamics and the functional elements of the genome. Alternative splicing (AS) is one of the most conventional posttranscriptional cellular mechanisms generating multiple isoforms from a single precursor mRNA (pre-mRNA) by the combination of different splice sites during messenger RNA (mRNA) processing. AS of pre-mRNA contributes to the complexity and diversity of the transcriptome and proteome in all eukaryotic organisms (Stamm et al. 2005). Several AS events such as exon skipping, alternative donor splice sites or acceptor sites, mutually exclusive exon, and intron retention are predominantly observed in eukaryotic organisms. Nearly 95% of multi-exon genes in the human genome undergo AS (Pan et al. 2008) while 61% have been shown to display AS in Arabidopsis (Marquez et al. 2012) and 33% in rice (Marquez et al. 2012; Zhang et al. 2010). Intron retention AS event is dominant in plants while exon skipping AS event occurs mostly in animals (Barbazuk et al. 2008).

The early approaches to transcriptome analysis include analysis of expressed sequences tags (ESTs) (Adams et al. 1991), Northern blotting and PCR aided transcript titration assay (PATTY) (Alwine et al. 1977; Becker-André and Hahlbrock 1989), serial analysis of gene expression (SAGE) (Velculescu et al. 1995), and DNA microarray (or “chip”) (Lockhart et al. 1996). Recently developed high-throughput next-generation sequencing technologies have superseded the earlier technologies and made transcriptome analysis more robust. The second-generation sequencing technology of RNA sequencing (RNA-Seq) has been widely adopted for the quantification of transcripts and alternative splicing isoforms. However, the inherent limitations of RNA-Seq, especially the short read lengths, make it poorly suited to assembly and determination of transcripts from complex genomes, gene isoform detection, and methylation detection (Rhoads and Au 2015; Wang et al. 2019, 2016). Long-read sequencing technology overcomes many of these limitations and enables the capture of full-length complementary DNA (cDNA) sequences and analysis of allele-specific expression (Rhoads and Au 2015). The Single-Molecule Real-Time (SMRT) Isoform-Sequencing (Iso-Seq) analysis in Pacific Biosciences (PacBio) based transcripts profiling allows multiple full-length transcripts to be identified without the possibility of erroneous computational assembly (Abdel-Ghany et al. 2016; Wang et al. 2019, 2016). Iso-Seq produces a single transcript up to 10 kb or longer characterizing the full diversity of isoform of a gene of interest. It covers all reads from the 5′ end to 3′ polyadenylic acid (poly-A) tail and, therefore alternatively spliced exons, transcription start sites and polyadenylation sites can easily be obtained. Iso-Seq has scaled up downstream analysis by generating high-quality full-length transcripts, which facilitate our understanding of posttranscriptional events such as alternative splicing (AS), alternative polyadenylation (APA), fusion transcripts, long non-coding RNAs, isoform phasing, genome annotation, and gene expression. The PacBio Sequel system with the latest version of the pipeline (Iso-Seq3) has incorporated faster clustering algorithm compared to the PacBio RSII system to generate high-quality polished transcripts and a higher number of perfectly annotated isoforms (Wang et al. 2019). PacBio RSII system has been widely used in the transcriptomics studies of many plants such as maize (Wang et al. 2016), coffee (Cheng et al. 2017), wheat (Dong et al. 2015), sugarcane (Hoang et al. 2017), cotton (Li et al. 2019), rice (Zhang et al. 2019), hot pepper (Zhu et al. 2018), and the rubber tree (Chow et al. 2019).

Rice has the smallest genome size (~ 430 Mbp) (Feng et al. 2002) compared to the other major cereal crops such as sorghum ~ 730 Mbp (Paterson et al. 2009), maize 4200 Mbp (Haberer et al. 2005), barley 5100 Mbp (Mayer et al. 2012), and wheat ~ 17,000 Mbp (Shi and Ling 2018). The genome of *Oryza sativa* L. ssp. *japonica* cv. Nipponbare was first sequenced and assembled using the whole-genome shotgun sequencing, capturing 93% of the genome (Goff et al. 2002). Later, a revised and error-corrected high-quality map-based reference genome sequence of *O. sativa* ssp. *japonica* cv. Nipponbare was developed in 2005 using a clone-by-clone sequencing strategy after construction of a minimum tiling path (MTP) for each chromosome and validating the final MTP with optical mapping data. Then fine-scale validation was done with error correction through second-generation re-sequencing data (Illumina Genome Analyzer II/IIx and Roche GS FLX) (Kawahara et al. 2013). The success of functional genomics studies relies on a profound and thorough understanding of the rice transcriptome (Zhang et al. 2010). The rice transcriptome has been studied in detail with over 28,000 full-length cDNA clones of japonica rice being used to elucidate the gene structure, in terms of, exon–intron boundaries and gene coding regions within genomic sequences (Kikuchi et al. 2003). In 2006, Wang and Brendel conducted ETS/cDNA-based analysis in rice to identify alternative splicing events. They mapped 298,857 ESTs and 32,136 full-length cDNAs obtained from GenBank to their respective genome sequences. Later, the RNA-Seq method has been used to capture a substantial number of novel transcripts, exons, and untranslated regions (UTRs) with the characterization of the alternative splicing events in the indica rice genome (Lu et al. 2010; Zhang et al. 2010). Furthermore, the transcriptome from japonica rice seedlings has been characterized by long-read sequencing to determine the number of spliced isoforms and transcriptome diversity due to AS and APA using a PacBio RSII platform (Zhang et al. 2019). As the improving sequencing technologies enable the capture of a wider array of high-quality full-length transcripts, it is possible to further investigate alternative splicing patterns, novel splicing isoforms, and coding regions potential of novel transcripts to improve the annotation of the rice genome for a wider range of tissues.

Here, we report the transcriptome of rice (*Oryza sativa* L. ssp. *japonica* cv. Nipponbare) from five organs including panicles, leaves, roots, inflorescences and developing seeds using PacBio long reads from a PacBio Sequel platform. This study uncovered a higher number of full-length transcripts than previously reported from seedlings of *O. sativa* L. ssp. *japonica* cv. Nipponbare using a PacBio RSII platform (Zhang et al. 2019). We also characterize alternative splicing isoforms with different lengths and splicing junctions. This study unveils a substantial number of novel isoforms of AS genes which may help to improve the current rice genome annotation.

## Materials and Methods

### Sample Collection

Five organs of rice (*O. sativa* ssp. *japonica* cv. Nipponbare); leaves, panicles, inflorescences, roots, and seeds were collected from plants grown in a glasshouse at the University of Queensland, Australia. Seeds and whole panicles were collected from six different development stages corresponding to 5, 10, 15, 20, 25, and 30 days postanthesis (DPA). Samples were snap-frozen with liquid nitrogen in the glasshouse immediately after collection and then stored at − 80 °C in a freezer. Total RNA was extracted separately from leaves, panicles, inflorescences, roots, and seeds using the RNA extraction protocol described by Furtado (2014). RNA was purified using the Qiagen RNeasy Mini Kit (Qiagen, Valencia, CA, United States). The purity, quality and quantity of isolated RNA were assessed by the 260/280 ratio on the NanoDrop 8000 Spectrophotometer (ThermoFisher Scientific, Waltham, MA, USA), the RNA integrity number (RIN) using an Agilent RNA 6000 Nano Kit on the Bioanalyzer 2100 (Agilent Technologies, Santa Clara, CA, USA), and the mass using the Qubit RNA BR assay kit on the Qubit 3.0 Fluorometer (ThermoFisher Scientific, Waltham, MA, USA), respectively. The RNA purity was calculated from the 260/280 ratio at absorbance 260 nm being around 2.0, and the RIN of the samples ranged between 6.20 and 9.70, indicating that RNA samples were suitable for sequencing.

### Preparation of cDNA PacBio Libraries

Equal mass of the total RNA samples was pooled. Aliquots of 900 ng input were prepared for the Iso-Seq cDNA library, as per the procedures for ‘Iso-Seq template preparation for Sequel Systems with size selection’ (PacBio, PN. 101-070-200, version 6, September 2018). First-strand of cDNA was synthesized with the SMARTer (Switching Mechanism at 5′ End of RNA Template) PCR cDNA synthesis kit (Takara Bio, San Jose, CA, USA, ref. 634926). High-quality double-stranded cDNA was amplified by the optimized 12-cycles large-scale PCR, using the high-fidelity PrimeSTAR GXL DNA polymerase (Takara Bio, San Jose, CA, USA, ref. R060A). For size selection, the PCR products were pooled and divided into three fractions. Two of these fractions were processed and equimolar pooled for the ‘non-size selected’ template. The third fraction, designated for the ‘ > 4 kb sized’ template, was electrophoresed through a 0.75% agarose gel cassette for DNA size selection (Sage Science ref. BLF7503) on the BluePippin DNA Size-Selection System (Sage Science Inc., Beverly, MA, USA, ref. 978-9222-1832), with the collection threshold set to start at 3500 bp and ended at 20,000 bp. The Iso-Seq templates were prepared using the SMRTbell Template Prep Kit 1.0 (Pacific Biosciences, Menlo Park, CA, USA, ref. 100-259-100). The two templates were kept separate for the DNA damage and end repairs, hairpin adaptors blunt ligation, and exonuclease clean-up. Throughout the protocol, purification and size-selected clean-up were achieved with specified concentrations of AMPure PB magnetic beads (Beckman Coulter, Pasadena, CA, USA, for PacBio ref. 100-265-900). Quality control was performed using Qubit dsDNA HS and BR Assay kits with the Fluorometer for quantitation, and Agilent DNA 12,000 assay kits on the Bioanalyzer 2100 to determine DNA fragment sizes. The ‘non-size selected’ and ‘ > 4 kb sized’ SMRTbell templates were pooled at a 5:1 molar ratio, with 4pM diffusion loaded into the SMRT cell 1M v3 LR (PacBio, ref. 101-531-001), and sequenced with a movie collection time of 1200 min on a PacBio Sequel System.

### Iso-Seq Data Processing

The raw data from the PacBio Iso-sequencing were processed using the default parameters of the IsoSeq3 analysis application in SMRT Link V. 6.0 (https://www.pacb.com/videos/tutorial-iso-seq-analysis-application-smrt-link-v6-0-0/). The processing steps included generating circular consensus sequences (CCS), trimming, refining, clustering, and polishing. The following main parameters were applied: a minimum number of passes = 1, minimum predicted accuracy of subreads = 0.75, minimum predicted accuracy of reads = 0.8, no polishing after constructing initial templates, minimum accuracy of polished isoforms = 0.82.

Consensus sequences of a minimum of two full-pass subreads in a single zero-mode waveguide (ZMW) were used to produce a circular consensus sequence (CCS). Based on the presence of the 5′ primer and 3′ primer or 3′ terminal poly-A tail, reads were classified into full-length (FL), or non-full length (NFL). Reads containing 5′ and 3′ cDNA primer, and a poly-A tail were considered full-length non-chimeric (FLNC) reads. When ROIs (read of inserts) lack any of those tags classified as non-full length (NFL) reads. Trimming of FLNC lengths was then performed to remove barcoded, unbarcoded cDNA primers and unwanted primers, and turn the reads to the 5 → 3 orientation. Before generating clustered consensus sequences, FLNC reads were refined by trimming poly-A tails and removing artificial concatemers. Iterative clustering and error correction (ICE) algorithm was applied to generate clustered consensus sequences. A polishing arrow model, including Quiver (QV) track, was applied to polish multiple FLNC reads generated from identical isoforms to obtain non-redundant isoform sequences. The final FLNC transcripts sequences were further divided into high-quality (HQ) transcripts with ≥ 99% post-correction accuracy and low-quality (LQ) transcripts with < 99% accuracy.

### Transcriptome Characterization

The SQANTI3 (Structural and Quality Annotation of Novel Transcripts Isoforms) version 4.2 pipeline is designed to identify structural and quality annotation of novel transcript isoforms (Tardaguila et al. 2018). This pipeline was applied to characterize isoforms at both the transcript and splice junction levels. Default parameters of the SQANTI3 pipeline were applied, except for the open reading frame (ORF) option which was skipped. The high-quality reads (HQ) were first aligned with minimap2 using the Os-Nipponbare-Reference-IRGSP-1.0 downloaded at RAP-DB (Rice Annotation Project Database) (Kawahara et al. 2013). The SQANTI3 pipeline was used to compare each HQ read to the reference genome annotation at the locus where it mapped and estimate the number of annotated and novel genes. The SQANTI3 pipeline was also used to classify the spliced isoforms based on their splice junctions and the reference genome annotation as well as the structure of the splice variants.

### Characterization of Spliced Variants for Starch Synthesis Related Genes

As an example, the isoforms of 19 starch synthesis-related genes were evaluated using the SQANTI3 pipeline. The genes were *AGPL2* (*ADP-glucose pyrophosphorylase large subunit 2*), *GBSSI* (*Granule bound starch synthase I*), *GBSSII* (*Granule bound starch synthase II*), *SSI* (*Soluble starch synthase I*), *SSIIa* (*Soluble starch synthase IIa*), *SSIIb* (*Soluble starch synthase IIb*), *SSIIc* ((*Soluble starch synthase IIc*), *SSIIIa* (*Soluble starch synthase III-2*), *SSIIIb* (*Soluble starch synthase III-1*), *SSIVa* (*Soluble starch synthase IV-1*), *SSIVb* (*Soluble starch synthase IV-2*), *BEI* (*Starch branching enzyme I*), *BEIIa* (*Starch branching enzyme IIa*) *BEIIb* (*Starch branching enzyme IIb*), *ISA1* (*Isoamylase 1*), *ISA2* (*Isoamylase 2*), *PUL* (*Pullulanase*), *GPT1* (*Glucose-6-phosphate translocator*), and *starch phosphorylase* (*PHOL*). The different spliced isoforms of each starch synthesis-related gene were determined from SQANTI3 output. The structure of the isoforms of the starch synthesis-related genes was also determined from the SQANTI3 output and further validated through Integrated Genomics Viewer (IGV) version 2.11.2 software (Robinson et al. 2011).

### Alternative Splicing (AS) Junctions and AS Events

SQANTI3 pipeline analyses HQ transcripts based on their splice junctions and donor and acceptor sites. Based on the presence of two pairs of dinucleotides at the start and end of the introns, splice junctions were classified into canonical (i.e., GT-AG, GC-AG, and AT-AC) and non-canonical (i.e., other possible combinations). Known splice junctions were those present in the reference transcripts, however, if not present in the reference, transcripts were labelled as novel.

The annotated HQ transcripts (.gtf file) from the mapping output using the SQANTI3 pipeline was applied in AStalavista (alternative splicing transcriptional landscape visualization tool) version 5 software (Foissac and Sammeth 2007) to classify AS events. AS events were determined from a genomic annotation of exon–intron gene coordinates by comparing all the transcripts. Four major AS types, namely intron retention, exon skipping, alternative acceptor splice sites, and alternative donor splice sites were obtained from the output files and counted, respectively.

### Functional Annotation of Transcripts

The Blast2GO functional annotation workflow was applied to the Iso-Seq transcripts using OmicsBox version 1.3.11 (https://www.biobam.com/omicsbox/) to annotate the transcripts. To find homologous sequences of the query transcripts, non-redundant FLNC sequences (HQ 33,504 and LQ 152 sequences) were first blasted against the non-redundant protein sequences (nr v5) database derived from NCBI using the blastx-fast program with a blast expectation value (e-value) of 1.0E-10. While performing the blastx-fast search, sequences were blasted against the Viridiplantae database (https://www.ncbi.nlm.nih.gov/Taxonomy/Browser/wwwtax.cgi?mode=info&id=33090) derived from NCBI. The BLAST (Basic Local Alignment Search Tool) results were subjected to gene ontology (GO) mapping (Götz et al. 2008) and annotation. The Cloud InterProScan was applied to search for protein domains from the EMBL-EBI (EMBL's European Bioinformatics Institute) databases. All GO terms retrieved via the InterProScan were then merged to the GO annotation derived after GO mapping to improve annotation and to retrieve reliable plant generic GO terms. Transcription factors (TFs) were determined by annotating transcripts against the plant transcription factor database (PlantTFDB version 5.0) (Jin et al. 2017). TFs obtained from the annotated genome of *O. sativa* ssp. *japonica* var. Nipponbare (MSU Rice Genome Annotation Project Release 7; http://rice.uga.edu/) available on plant transcription factor database (Jin et al. 2017) were also compared with the TFs determined using PacBio Iso-Seq data. To investigate plant-related metabolic and biological pathways that are linked to the transcript sequences, the Kyoto Encyclopedia of Genes and Genomics (KEGG) (Kanehisa et al. 2016) pathways were determined in OmicsBox 1.3.11. software using the KEGG pathway tool with default parameters.

### Non-coding Transcripts

Iso-Seq sequences without BLAST hits to NCBI-nr were submitted to the Rfam 14.7 database for long non-coding RNA (lncRNA), microRNA (miRNA), and other types of RNAs annotation using OmicsBox 1.3.11(https://www.biobam.com/omicsbox/). Then sequences without a Rfam hit were deployed to the coding potential calculator 2 (CPC2) web server (Kang et al. 2017) to predict the coding ability of transcripts along with the length of peptide homologous to the open reading frames (ORFs). The protein-coding transcripts usually contain a long and high-quality ORF compared to the non-coding transcripts. At the RNA level, ORF length and integrity are two powerful measures as protein-coding transcripts usually contain a long and high-quality ORF compared to non-coding transcripts (Kang et al. 2017). Therefore, only ORF length and coding probability score were considered for determining the non-coding transcripts. CPC2 generates two types of putative ORF integrity: complete ORF and incomplete ORF. CPC2 score < 0.5 is regarded as a non-coding transcript (Kang et al. 2017). If the ORF length is < 200 amino acids (aa) it is regarded as small non-coding RNA (sncRNA) while the ORF ≥ 200 aa is regarded as lncRNA (Cao 2014; Mattick 2001). Furthermore, these potential non-coding RNAs were submitted to well-annotated databases, including Swiss-Prot (Boutet et al. 2016), RNAdb (Pang et al. 2007), and lncRNAdb (Quek et al. 2015) to search for homology sequences to validate the findings from CPC2 web server.

### Coding Potential of HQ Transcript Isoforms

To determine putative protein-coding regions within the Iso-Seq transcript sequences, the predict coding regions tool based on TransDecoder 5.5.0 software (Haas et al. 2013) in OmicsBox 1.3.11 was applied to predict open reading frames (ORFs) with a minimum protein length = 51 bp. The captured ORFs are scanned for homology to known proteins through the Pfam database (Punta et al. 2012) to identify common protein domains. The predicted ORFs encoded for proteins of four types based on start and stop signals: complete with both start and stop codon, 3′partial with missing stop codon and presumably part of the C-terminus, 5′partial with missing start codon and presumably part of the N-terminus (s), and internal with both start and stop codons missing.

## Results

### The Output of PacBio Sequel Sequencing

A total of 16,551,194 subreads from the cDNA library were generated after removing the adapters from 517,297 polymerase reads with an average length of 65,287 bp (Table 1). A total of 415,221 CCS reads was obtained from these subreads using the IsoSeq3 analysis application. After removing barcoded and unbarcoded cDNA primers, a total of 357,925 full-length sequences were identified. Of these, a total of 346,190 non-redundant FLNC sequences were generated after trimming the poly-A tail and clustering with the iterative clustering and error correction (ICE) algorithm. A total of 33,658 unpolished FLNC reads were determined with the length of the longest read 14,636 bp. For polished clustering of the FLNC reads, the arrow model with the Quiver algorithm was applied to generate isoforms that were classified as high quality (HQ) and low quality (LQ) isoforms based on the estimated accuracy. An estimated Quiver accuracy < 99% is classified as LQ isoforms and HQ isoforms show ≥ 99% accuracy. A total of 33,504 HQ isoforms and 152 LQ were obtained with an average length of FLNC reads of 2971.10 bp (Additional file 3: Fig. S1).

**Table 1:** PacBio SMRT sequencing data

Category	Dataset
Polymerase reads	517,297
Mean length of polymerase	65,287 bp
Polymerase reads N50	134,505
Number of subreads	16,551,194
Mean length of subreads	1997 bp
Number of the circular consensus sequences (CCS) reads	415,221
Number of full-length (FL) reads	357,925
Number of full-length non-chimeric (FLNC) reads	346,190
Number of unpolished FL reads	33,658
Length of the longest unpolished FL reads	14,636 bp
Number of high quality (HQ) FLNC reads	33,504
Number of low quality (LQ) FLNC reads	152
Mean FLNC reads length	2971.10 bp

### Characterization of PacBio Iso-Seq Transcripts

**Fig. 1:**
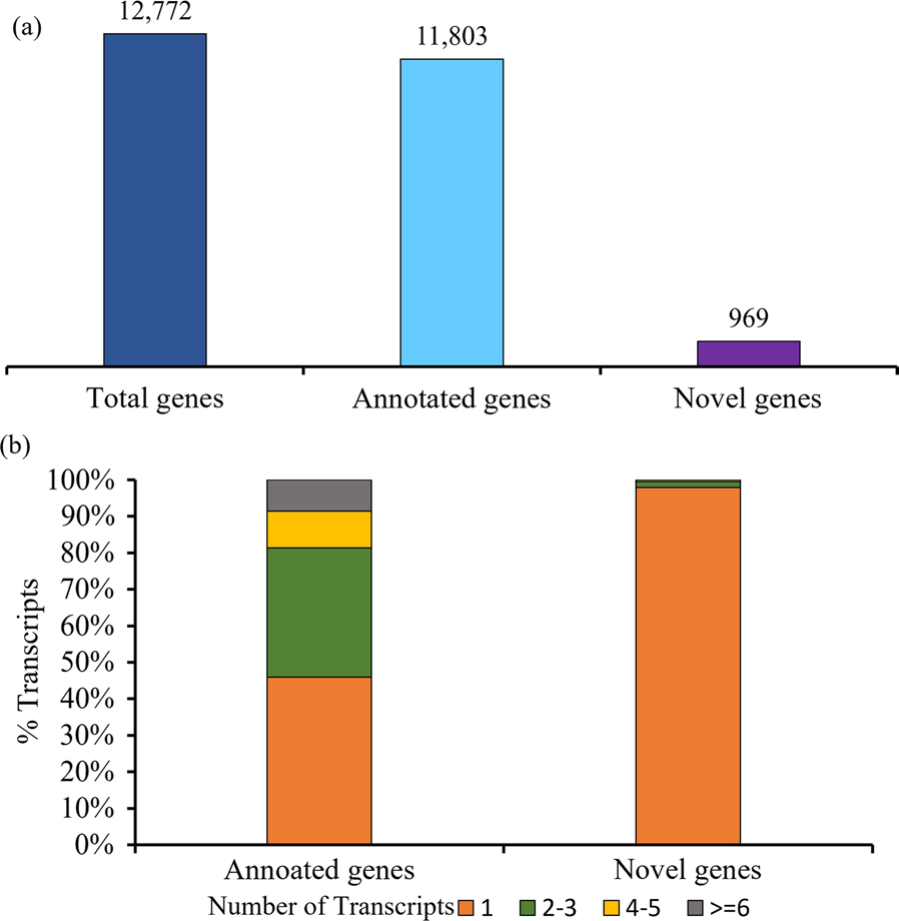
PacBio transcriptome characterization: **a** quantification of total genes comprising of annotated genes and novel genes; **b** transcripts distribution across the annotated and novel genes. Annotated genes: annotated to the reference genome; Novel genes: not annotated to the reference genome

SQANTI3 is a pipeline for the in-depth characterization of isoforms acquired by full-length transcripts sequences. SQANTI3_qc.py (Tardaguila et al. 2018) was employed to investigate the number of unique genes and isoforms after mapping the high-quality consensus sequences (33,504) to the Os-Nipponbare-Reference-IRGSP-1.0 reference genome (Kawahara et al. 2013). The number of total genes was 12,772 corresponding to a total of 33,456 transcript isoforms. Of these genes, 11,803 genes were annotated with the reference transcripts. While 969 novel genes had no annotation information from the reference transcripts (Fig. 1a). These novel genes originated from transcripts lying outside of annotated genes and poly-A containing transcripts overlapping the complementary strand of an annotated transcript. The transcript types of annotated and novel genes were mainly categorized as multi-exon and mono exon. Multi-exon transcripts in annotated genes and novel genes accounted for 87% (10,259) and 38% (370) respectively. Mono-exon transcripts in novel genes represented 62% (599) while 13% (1544) of mono-exon transcripts had an annotation. The number of isoforms per gene ranged from 1 to ≥ 6 (Fig. 1b). Most of the annotated genes (5424 genes) with a single transcript (45.95%) followed by 35.39% (4177 genes) of annotated genes with 2–3 isoforms, 10.06% (1187 genes) with 4–5 transcripts, and only 8.6% (1015 genes) with equal to or more than 6 transcripts. In contrast, 97.52% (945 genes) of novel genes had a single transcript per gene while 1.55% (15 genes) and 0.52% (5 genes) of novel genes were composed of 2–3 transcripts and 4–5 transcripts per gene, respectively. Surprisingly, only 0.41% (4 genes) of novel genes contained ≥ 6 transcripts per gene.

### Classification of Isoforms

To characterize isoforms, the SQANTI3 pipeline classifies each HQ transcript into the following categories based on a comparison between the splice junctions and the reference genome annotation: Full Splice Match (FSM), Incomplete Splice Match (ISM), Novel In Catalog (NIC), Novel Not in Catalog (NNC), Intergenic (i.e., a novel isoform without any previously annotated genes in the annotated reference genome), Genic intron (i.e., a novel transcript residing entirely within the boundaries of an annotated intron), Genic genomic (i.e., a novel isoform that overlaps annotated exons and introns), Fusion (i.e., a novel isoform that lies on two different annotated loci in the annotated genome), and Antisense (i.e., a novel isoform containing poly-A, which matches a reverse strand of an annotated transcript).

FSM explains transcripts that are fully matched with reference transcripts at all splice junctions. ISM are those transcripts that miss out on exons at either the 3′ or 5′ end or both ends of the transcript resulting in incomplete matches at all splice sites. UTR3 fragments result from ISM transcripts which have more than 95% of their sequences within the UTR3 sequence of their related reference genome. NIC transcripts represent novel isoforms with another combination of already known exons but with a combination of known splice junctions from already annotated isoforms. While NNC novel isoforms are not present in the annotation and therefore, these have at least one novel exon or intron retention.

**Fig. 2:**
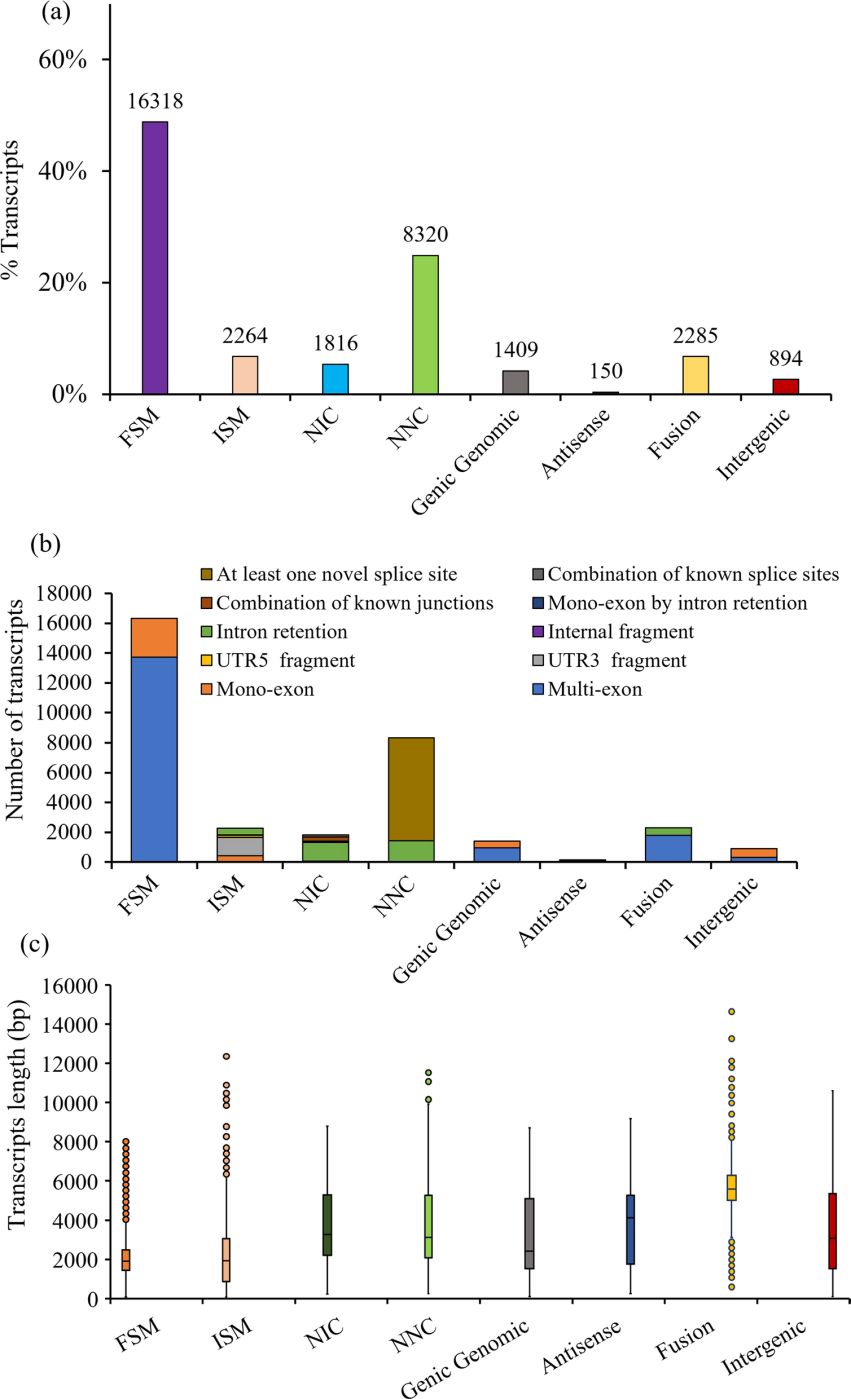
Characterization of isoform categories of PacBio transcriptome: **a** percentage of the PacBio transcriptome in different isoform categories (*FSM* full splice match, *ISM* incomplete splice match; *NIC* Novel In Catalog, *NNC* Novel Not in Catalog, Genic Genomic, Antisense, Fusion, and Intergenic); **b** structure of different categories of isoforms **c** length of PacBio transcripts by different isoform categories

According to SQANTI3 classification, FSM and ISM transcripts when mapped to Os-Nipponbare-Reference-IRGSP-1.0 reference transcripts exons represented 48.8% (16,318) and 6.8% (2264) of the transcriptome, respectively (Fig. 2a). NIC and NNC that were novel isoforms of known genes accounted for 5.4% (1816) and 24.9% (8320), respectively. Moreover, genic genomic, antisense, fusion, intergenic categorized as novel isoforms represented 4.2% (1409), 0.4% (150), 6.8% (2285), and 2.7% (894) of the total, respectively. The structure of isoforms distinctly differed in different isoform categories (Fig. 2b). FSM isoforms were mostly composed of multi-exon (13,725) and 2593 transcripts were mono-exon transcripts. While ISM isoforms were characterized as mono-exon (428), UTR3 fragment (1229), UTR5 fragment (150), Internal fragment (15), and Intron retention (442). Novel isoforms (NIC) showed a range of unannotated configuration in an annotated exons/splice site: mono-exon (50), intron retention (1264), mono-exon by intron retention (77), a combination of known junctions (306), and a combination of known splice sites (119). Novel isoforms (NNC) were composed of intron retention (1438) and at least one novel splice site (6882). Genic genomic, Antisense, and Intergenic type of isoforms were represented by both multi- and mono exon. Fusion isoforms were made up of multi-exon (1786) and intron retention (499) isoforms. Differences in the structures of isoform categories led to remarkedly variable isoform length (Fig. 2c). ISM isoforms that matched the reference transcripts were longer transcripts than the FSM isoforms. The length of the longest ISM transcripts was 12,355 bp compared to that of FSM (8008 bp). This might be because the UTR3 fragments matched the longest reference transcripts, which contributed to the longest ISM transcript. Among novel isoforms categories, NNC transcripts (11,667 bp) were longer than NIC transcripts (8802 bp) due to an elevated number of novel splice sites. The other classes of novel isoforms (genic genomic, antisense, and intergenic), except for fusion showed almost similar transcript lengths. However, fusion transcript lengths were noticeably greater than those of ISM as fusion was not made up of any mono-exons. The transcript length of fusion isoforms ranged between 589 bp and 14,645 bp.

### Characterization of Isoforms for Starch Synthesis Related Genes

Isoforms corresponding to starch synthesis-related genes were determined from the SQANTI3 output as an example to understand the complexity of rice transcriptome. A total of 276 isoforms were found for starch synthesis-related genes (Table 2). In the ADP-glucose pyrophosphorylase enzyme group, *AGPL2* had 19 spliced isoforms with the largest transcript length of 2459 bp. The starch synthase enzyme group was separated into two types: granule-bound starch synthase (GBSS) and soluble starch synthase (SS). Two genes from the GBSS group of enzymes, namely *GBSSI* and *GBSSII* represented 28 and 19 isoforms in which the longest transcript length was observed in *GBSSI* (3615 bp). *SSIIIa* accounted for the highest number of isoforms (51) with the largest transcript length (7791 bp) in the soluble starch synthase enzyme group, followed by 9 isoforms for *SSI* and *SSIIa* in each, 7 isoforms for *SSIIIb*, 5 isoforms for *SSIIb,* 3 isoforms for *SSIIc,* 1 isoform for *SSIVa*, and 2 isoforms for *SSIVb*. In the starch branching enzyme group, *BEI* was represented by 45 isoforms, followed by 5 isoforms for *BEIIa*, and 28 isoforms for *BEIIb*. However, the transcripts lengths for *BEI* and *BEIIb* were almost the same. Among the three starch debranching genes, *PUL* had the highest number of isoforms (15), more than that of *ISA1*(8) and *ISA2* (4). Likewise, *PHOL* accounted for 15 isoforms while *GPT1* had only 3 isoforms.

**Table 2:** Distribution of isoforms with length (in bp) in 19 starch synthesis related genes

Starch synthesis related genes	Total number of isoforms	Transcript length (bp)	Isoform categories					
			FSM	ISM	NIC	NNC	Fusion	Genic genomic
*AGPL2*	19	261–2459	–	5	–	14	–	–
*GBSSI*	28	86–3615	12	10	1	5	–	–
*GBSSII*	19	2039–2976	15	–	4	–	–	–
*SSI*	9	1764–2800	8	1	–	–	–	–
*SSIIa*	9	1480–2934	–	2	–	7	–	–
*SSIIb*	5	2385–2447	–	4		1	–	–
*SSIIc*	3	2888–3057	–	–	–	3	–	–
*SSIIIa*	51	2175–7791	34	4	3	3	7	–
*SSIIIb*	7	1978–5047	3	1	3	–	–	–
*SSIVa*	1	3643	1	–	–	–	–	–
*SSIVb*	2	3316–4797	1	–	1	–	–	–
*BEI*	45	127–5352	3	30	2	10	–	–
*BEIIa*	5	993–1208	5	–	–	–	–	–
*BEIIb*	28	143–5077	–	11	–	16	–	1
*ISA1*	8	2068–3010	6	2	–	–	–	–
*ISA2*	4	2619–2888	4	–	–	–	–	–
*PUL*	15	278–4771	–	2	–	13	–	–
*GPT1*	3	1723–1830	3	–	–	–	–	
*PHOL*	15	1203–3439	7	8	–	–	–	–

*AGPL2—ADP-glucose pyrophosphorylase large subunit 2*, *GBSSI—Granule-bound starch synthase I*, *GBSSII—Granule-bound starch synthase II*, *SSI—Soluble starch synthase I*, *SSIIa—Soluble starch synthase IIa*, *SSIIb—Soluble starch synthase IIb*, *SSIIc—Soluble starch synthase IIc*, *SSIIIa—Soluble starch synthase III-2*, *SSIIIb—Soluble starch synthase III-1*, *SSIVa—Soluble starch synthase IV-1*, *SSIVb—Soluble starch synthase IV-2*, *BEI—Starch branching enzyme I*, *BEIIa—Starch branching enzyme IIa*, *BEIIb—Starch branching enzyme IIb*, *ISA1—Isoamylase 1*, *ISA2—Isoamylase 2*, *PUL—Pullulanase*, *GPT1—Glucose-6-phosphate translocator*, *PHOL—Starch phosphorylase*

*FSM* full splice match, *ISM* incomplete splice match, *NIC* Novel In Catalog, *NNC* Novel Not in Catalog

Of eight categories of isoforms, six isoform types (FSM, ISM, NIC, NNC, Fusion, and Genic Genomic) were identified for 19 starch synthesis-related genes (Table 2). A total of 102 FSM type of isoforms were determined corresponding to *GBBSI* (12), *GBSSII* (15), *SSI* (8), *SSIIIa* (34), *SSIIIb* (3), *SSIVa* (1), *SSIVb* (1), *BEI* (3), *BEIIa* (5), *ISA1* (6), *ISA2* (4), *GPT1* (3), and *PHOL* (7). In the ISM type of isoforms, the highest number of isoforms was found in the starch branching group of enzymes (*BEI* and *BEIIb*) which accounted for 30 and 11 isoforms, respectively. Granule-bound starch synthase gene (*GBSSI*) represented 10 ISM isoforms. A total of 12 ISM isoforms were determined for the soluble starch synthase group of genes: *SSI* (1), *SSIIa* (2), *SSIIb* (4), *SSIIIa* (4), and *SSIIIb* (1). Only 2 ISM isoforms were determined in each of *ISA1* and *PUL* starch debranching genes. Furthermore, *AGPL2* and *PHOL* accounted for 5 and 8 ISM isoforms. Unlike ISM, novel isoforms with annotated exons (NIC) represented 5 isoforms for granular starch synthase genes (*GBSSI* = 1 and *GBSSII* = 4), 7 isoforms for soluble starch synthase genes (*SSIIIa* = 3, *SSIIIb* = 3 and *SSIVa* = 1), 2 isoforms for starch branching genes (BEI). NNC categories of isoforms were determined in four classes of enzymes: 14 isoforms for ADP-phosphorylase enzyme (*AGPL2*), 19 isoforms for starch synthase (*GBSSI* = 5, *SSIIa* = 7, *SSIIc* = 3, *SSIIb* = 1, and *SSIIIa* = 3), 26 isoforms for starch branching group of enzymes ( *BEI* = 10 and *BEIIb* = 16), and 13 isoforms for starch debranching genes (*PUL*), The other novel categories of isoforms (Fusion and Genic Genomic) were determined in *SSIIIa* and *BEIIb* representing 7 isoforms and 1 isoform, respectively.

**Fig. 3:**
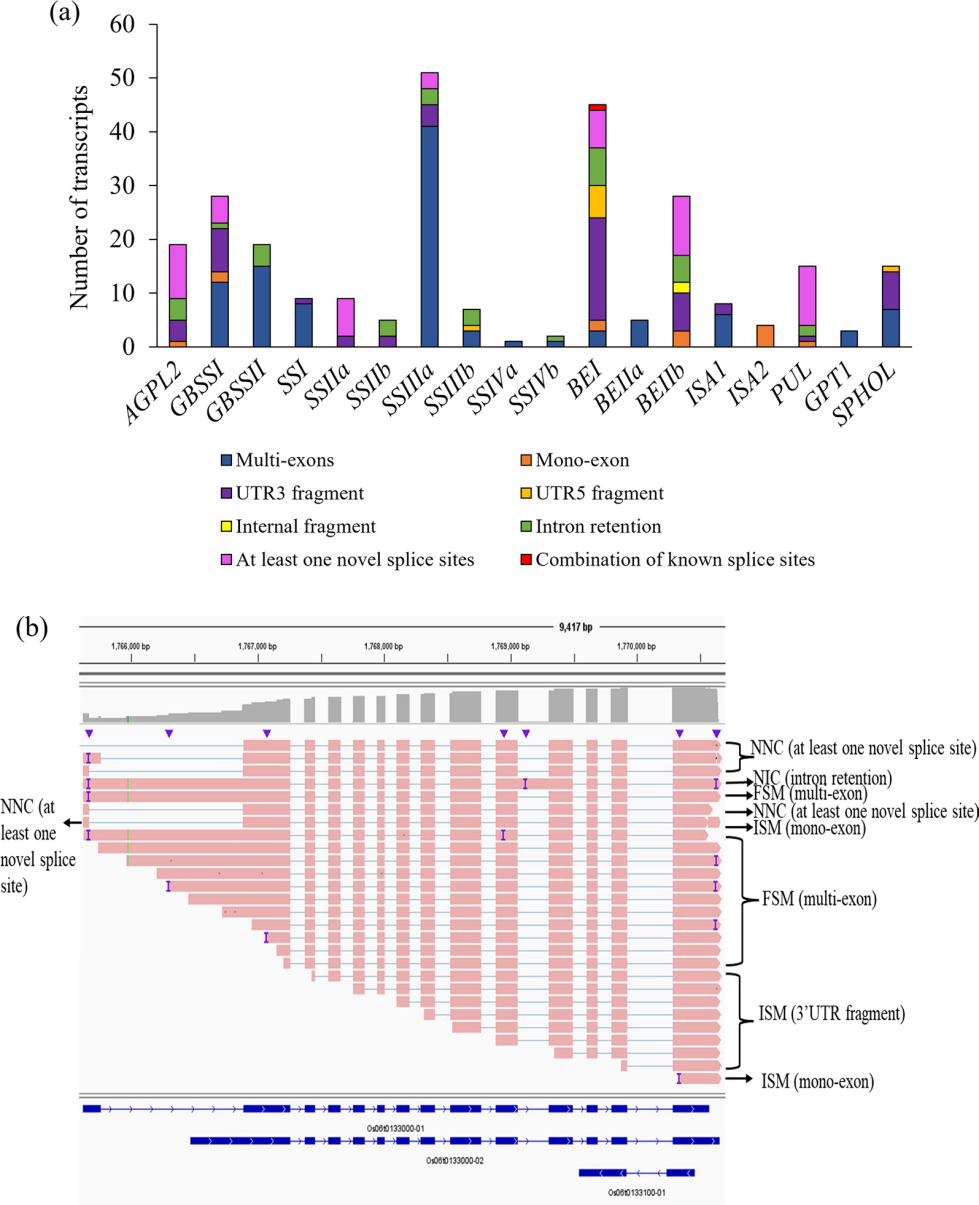
Structure of isoforms for starch synthesis related genes: **a** variation in the composition of isoforms of 19 starch synthesis related genes; **b** visualization of the 28 alternative splicing isoforms of the *GBSSI* (*granule-bound starch synthase I*) gene shown as an example. Grey panel: read coverage, Brown panel: splice junctions; Pink panel: alignment trach representing 28 spliced isoforms of the *GBSSI* gene; I: insertion; Blue arrow: indicate the position of insertion; Blue panel: reference transcripts of *GBSSI*

The structure of isoforms for starch synthesis-related genes was also determined from SQANTI3 scripts (Fig. 3a) and visualized via IGV (Fig. 3b). *AGPL2* isoforms were composed of mono-exon (1), UTR3 fragment (4), intron-retention (4), and at least one novel splice site (10). 28 isoforms of *GBSSI* represented 12 multi-exon, 2 mono-exon, 8 UTR3-fragment, 1 Intron retention, and 5 at least one novel splice site. While *GBSSII* isoforms were featured by only multi-exon (15) and Intron retention (4). In the soluble starch synthase group of enzymes, the highest number of multi-exons were observed in *SSIIIa* (41), followed by *SSI* (8), *SSIIIb* (3), *SSIVa* (1), and *SSIVb* (1). In addition, the UTR3 fragment was a feature of *SSI* (1), *SSIIa* (2), *SSIIb* (2), *SSIIIa* (4) while the UTR5 fragment in *SSIIIb* (1) was determined. Intron retention in *SSIIb* (3), *SSIIIa* (3), *SSIIIb* (3), *SSIVb* (1), and at least one splice site in *SSIIa* (7), *SSIIc* (3), and *SSIIIa* (3) were determined. Unlike, soluble starch synthase genes, *BEI* isoforms were mostly composed of UTR3 fragment (19), followed by Intron retention (7), at least one novel splice site (7), UTR5 fragment (6), multi-exon (3), mono-exon (2), and combination of known splice site (1). While *BEIIb* isoforms were mostly formed by at least one novel splice site (11) along with intron retention (5), internal fragment (2), UTR3 fragment (7), and mono exon (3). *BEIIa* isoforms were only made up of multi-exon (5). Starch debranching isoforms were formed by multi-exon, mono-exon, UTR3 fragment, UTR5 fragment, intron retention, and at least one novel splice site. *ISA1* isoforms were composed of 6 multi-exon and 2 UTR3 fragments while *ISA2* isoforms were represented by only 4 UTR3 fragments. *PUL* isoforms consisted of 1 mono-exon, 1 UTR3 fragment, 2 intron retention, and 11 at least one novel splice site. *GPT1* isoforms were only contained multi-exon (3) while *PHOL* isoforms were formed by multi-exon (7), UTR3 fragment (7), and UTR5 fragment (1). 28 isoforms of the *GBSSI* gene were visualized comprising of 12 FSM (12 multi-exon), 10 ISM (2 mono-exon and 8 UTR3-fragment), 1 NIC (1 Intron retention), and 5 NNC (5 at least one novel splice site) (Fig. 3b).

### Analysis of Alternative Splicing

**Fig. 4:**
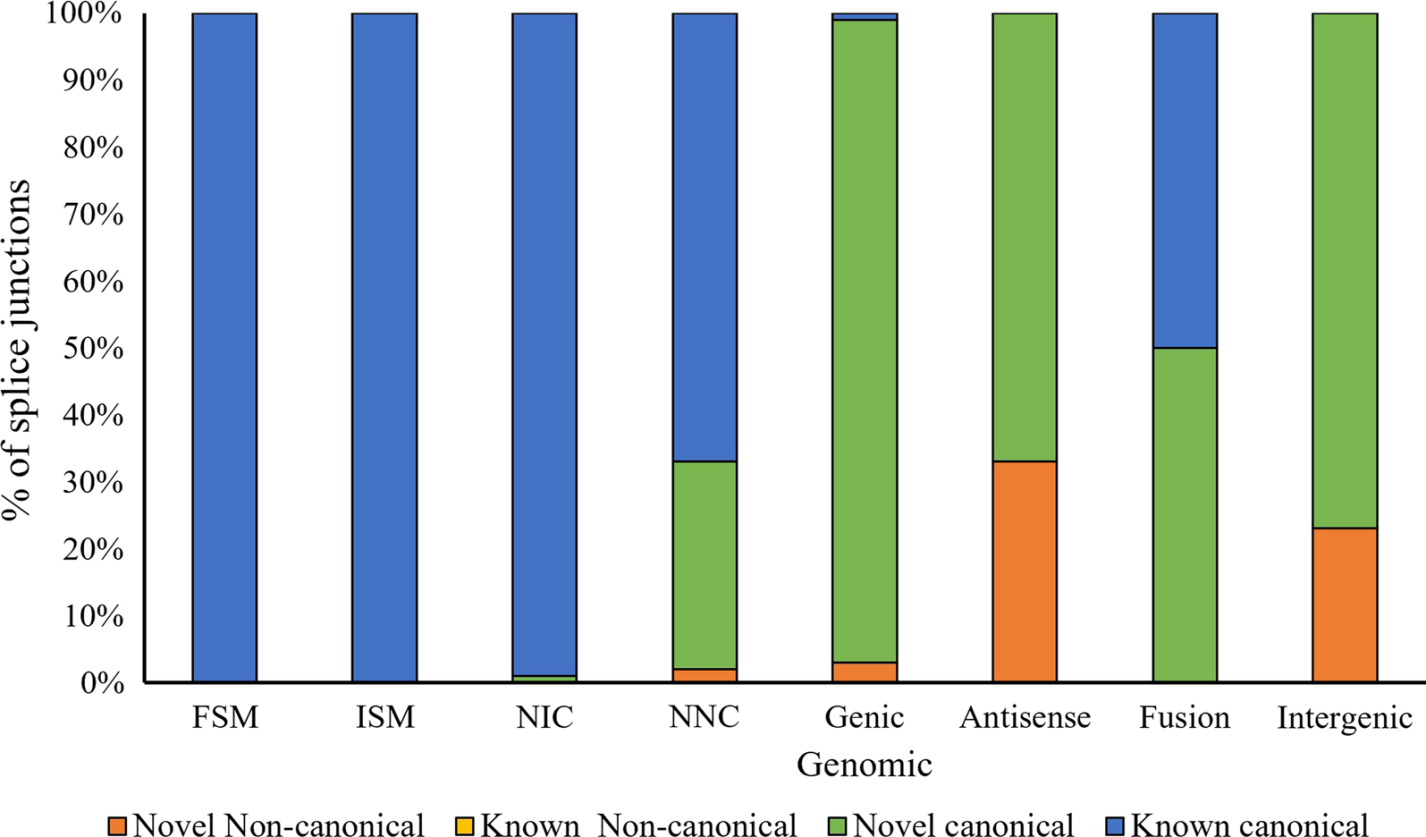
Distribution of alternative splice junction across all the isoform features

A total of 73,659 splice junctions were determined with SQANTI3 scripts. Out of 60,904 known splice junctions, 99.96% (60,878) were canonical (i.e., GT-AG, GC-AG, and AT-AC intron sites) and 0.04% (26) were non-canonical (i.e., other possible combinations). Splicing sites that are matched to the reference transcripts are regarded as known splice junctions. Similarly, out of 12,755 novel splice junctions, 11,812 (93%) of the splice junctions were canonical and only 943 (7%) were non-canonical. However, the distribution of known and novel splice junctions varied across the isoforms categories. All the known canonical splice junctions were FSM (100%) and ISM (100%) as both were derived entirely from known splicing events (Fig. 4). As NIC isoforms are made up of annotated splice sites in unannotated arrangement, 99% of the splice junctions were known canonical and only 1% were novel canonical splice junctions. In NNC, isoforms were made up of at least one unannotated splicing site and, therefore around 33% of the splicing isoforms were novel represented by both novel canonical and novel non-canonical. Furthermore, genic genomic, antisense, and intergenic transcripts were categorized as novel transcripts showing comparatively high percentages of novel canonical splice junctions with around 96%, 67%, and 77%, respectively. Fusion isoforms were made up of an equal percentage of known and novel canonical splice junctions, as fusion isoforms correspond with two different annotated loci. No known non-canonical splice junctions along with a low percentage of novel non-canonical splicing junctions inferred that those experimental artifacts might not influence these isoform categories.

HQ annotated transcripts were assessed to elucidate the molecular mechanisms (i.e., exon–intron boundaries) of alternative splicing events with AStalavista version 5 software. Of 6461 AS events, 2894 represented intron retention type of AS event followed by 248 AS events for exon slipping, 872 for alternative acceptor splice sites, 632 for alternative donor splice sites, and 1815 for other AS events (Additional file 1: Table S1). Intron retention was appeared to be the most dominant AS event.

### Functional Annotation of Iso-Seq Transcripts

**Fig. 5:**
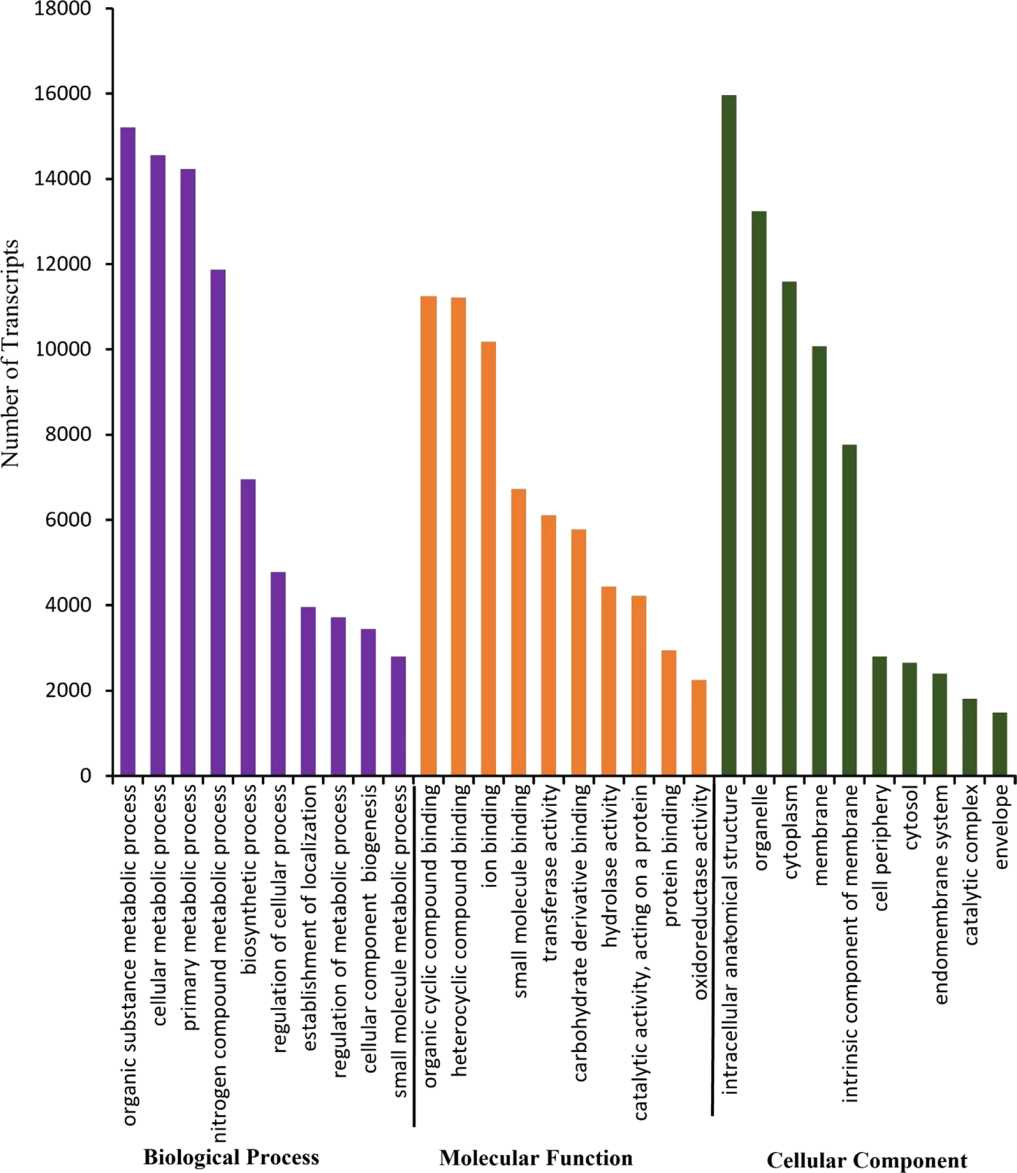
Top ten GO terms represented by the highest number of transcripts for cellular component, molecular function, and biological process categories of gene ontology annotation for all aligned transcript isoforms

A total of 33,656 transcript isoforms, including 33,504 HQ isoforms and 152 LQ isoforms were subjected to blast against the Cloudblast database. 32,880 transcript isoforms matched the NCBI-nr database of which 28,675 transcript isoforms were subjected to GO mapping. 28,487 transcript isoforms were annotated with GO terms. A total of 33,656 sequences were matched to InterProScan domains. When blasted against the Viridiplantae database, transcript isoforms showed similarity with 1424 plant species. Of these, the highest number of BLAST hits (100,793 hits) was observed for the *Oryza sativa* Japonica Group, followed by 33,080 hits for the *Oryza sativa* Indica group, and 32,503 hits for *Oryza sativa*. The gene ontology annotation was classified into three main categories: biological process (BP), molecular function (MF), and cellular component (CC) (Fig. 5). Of the 2488 biological processes, the highest number of isoforms showed the GO terms for organic substance metabolic process (15,212) followed by cellular metabolic process (14,555), and primary metabolic process (14,230). While the highest number of sequences (11,238) had GO terms for organic cyclic compound binding molecular function, followed by heterocyclic compound binding function (11,219) and ion binding function (10,184). Most of the biological processes and molecular function occurred in the intracellular anatomical structure (15,964), followed by 13,246 sequences for organelle and 11,590 sequences for cytoplasm.

From the plant transcription factor database, a total of 1367 TFs were identified in Iso-Seq transcripts, comprising of 52 TF families while 2408 TFs with 56 TF families were determined in *O. sativa* ssp. *japonica* (MSU_osa1r7) available on the plant transcription factor database (PlantTFDB version 5.0) (Jin et al. 2017) (Additional file 4: Fig. S2). The highest number of transcripts was identified in the MYB3-related family protein (106), followed by the C3H family protein (96), C2H2 family protein (89), NAC family protein (86), and bHLH family protein (71). Both PacBio Iso-Seq transcripts and (MSU_osa1r7) annotation generated 106 TFs for the MYB-related protein family while TFs corresponding to the CAMTA TF family was higher in the PacBio Iso-Seq transcripts (23 TFs) compared to *O. sativa* ssp. *japonica* (MSU_osa1r7) (7 TFs). Likewise, the number of TFs from Iso-Seq data was higher in the TF families such as HB-other (47), ARF (54), C3H (96), TALE (58), GATA (31), Co-like (22), and SBP (31).

To determine the metabolic and biological pathways represented by the largest number of Iso-Seq transcripts, KEGG pathway analysis was applied. Starch and sucrose metabolism (ko00500) was linked with the greatest number of transcript sequences (737), followed by spliceosome (ko03040) with 561 sequences, protein processing in the endoplasmic reticulum (ko04141) with 476 sequences, biosynthesis of cofactors (ko01240) with 460 sequences, RNA transport (ko03013) with 441 sequences, pyruvate metabolism (ko00620) with 393 sequences, plant hormone signal transduction (ko04075) with 383 sequences, glycolysis (ko00010) with 362 sequences, glyoxylate and dicarboxylate metabolism (ko00630) with 329, and plant-pathogen interaction (ko04626) with 328 sequences (Additional file 5: Fig. S3).

### Identification of Non-coding RNA

A total of 776 Iso-Seq transcripts did not match with the Cloudblast database. To identify non-coding RNA families (miRNA, lncRNA, and others), these sequences were blasted against the Rfam database. Iso-Seq sequences matched to one transfer RNA (tRNA) and two miRNA (MIR535 and mir-160) belonging to the MIR535 and mir-160 gene family. One annotated GO term (GO:0030533) was found for tRNA corresponding to molecular function (triplet codon-amino acid activity) while two GO terms (GO:0035068 and GO:0035195) were found for two miRNA corresponding to RNA-induced silencing complex (RISC complex) functioning in gene silencing. Sequences without Rfam hits (773) were submitted to CPC2 analysis to predict coding potential. Of 773 transcripts, 721 transcripts showed coding potential for non-coding RNAs including 551 with complete putative ORF and 170 with incomplete putative ORF (Additional file 2: Table S2 attached at the end of the manuscript). The peptide length of 715 non-coding RNAs with a complete putative ORF was 2–216 aa. Six incomplete non-coding RNAs were without a putative ORF. Those with an ORF length < 200 aa were regarded as small non-coding RNA (sncRNA) while those with ORF length ≥ 200 aa were regarded as lncRNA. Of 721 potential non-coding RNAs, only 3 lncRNAs were identified with a complete putative ORF (200–216) aa. These ncRNAs were novel as there were no homologous sequences found in the Swiss-Prot, RNAdb, and lncRNAdb database.

### Coding Potential of Isoforms

It is essential to validate the wide range of novel transcripts characterized from different splicing events from single-molecule long-read sequencing-based approaches as not all of them have a significant impact on the cellular and biological processes of the cell. Wang et al. (2019) have proposed that the validity of those isoforms can be determined based on a few conditions such as the presence of an open reading frame (ORF) and CDS (coding sequence) length, InterPro domain coverage, annotation edit distance, and their spatio-temporal expression levels. In this study, candidate coding regions within transcripts were evaluated based on their ORF to elucidate the effect of transcript isoforms on the coding regions. Four types of ORFs were determined: complete ORF with both start and stop codon, 3′partial ORF with missing stop codon and presumably part of the C-terminus, 5′partial ORF with missing start codon, and presumably part of the N-terminus, and internal ORF with missing both start and stop codon. A total of 62,981 ORFs were predicted from 33,456 HQ transcript isoforms. The majority of the predicted ORFs were complete ORF types (46,952), followed by 3′partial (1965), 5′partial (13,956), and internal (108) types. The CDS length of the complete ORFs ranged from (203–14,645) bp, followed by (181–10,159) bp for 3′partial ORFs, (169–12,355) bp for 5′partial ORFs, and (154–5618) bp for internal ORFs. The number of ORFs was higher than the query transcript isoforms because a single transcript can generate multiple ORFs allowing for operons, chimeras, etc. 11,076 transcripts of the total had multiple ORFs of which 1758 transcripts had both complete and 3′partial ORF and 9318 transcripts had both complete and 5′partial ORF. This result inferred that the isoforms determined from SQANTI3 scripts might not be artifacts and instead had coding potential.

## Discussion

This study reveals rice transcriptome complexity from five organs including roots, leaves, inflorescences, panicles and developing seeds of *O. sativa* ssp. *japonica* var. Nipponbare using a PacBio Sequel platform. The number of FLNC reads generated for a range of tissues in this study (346,190) is much higher than the number of FLNC reads (98,616) that were generated in an *O. sativa* ssp. *japonica* seedling transcriptome with a hybrid approach (STAIR) of PacBio RSII and RNA-Seq (Zhang et al. 2019). The average read length of FLNC was 2971.10 bp from the PacBio Sequel platform which was higher than found using a PacBio-RSII platform (average FLNC length 2576 bp for cDNA library with insert size > 3 kb) (Zhang et al. 2019). This will be a useful resource for rice genomics.

The transcriptome included 33,456 isoforms from 12,772 genes were determined. Of the total genes, 11,803 genes are annotated while 969 genes are novel. These annotated genes correspond to the genes identified (55,986) in Os-Nipponbare-Reference-IRGSP-1.0 release7 rice reference genome (Kawahara et al. 2013). The novel genes are mostly represented by a single transcript. Exons of 18,582 (55.54%) splicing isoforms are known to the reference transcripts while 14,874 (44.46%) splicing isoforms are novel due to the novel orientation of exons derived from alternative splicing junctions. A wide range of isoforms and splice junctions have been identified relative to the reference transcriptome. Around 49% (16, 318 FSM) of the total full-length HQ transcripts matched to the reference transcripts, comprising of 13,725 full-length multi-exon splicing isoforms. In this study, the Iso-Seq data generated a substantial number of novel spliced isoforms belonging to NIC, NNC, Antisense, Fusion, Intergenic, and Genic Genomic categories of isoforms. Antisense and Intergenic type of isoforms correspond to novel genes. Using the STAIR pipeline, 11,733 full-length multi-exon splicing isoforms were identified and validated of which 5204 spliced isoforms were not reported in the current reference transcriptome (MSU_osa1r7) and 4453 isoforms were absent in the rice transcriptome assembled by short RNA-Seq reads (Zhang et al. 2019). Deep RNA-sequencing at a single base-pair resolution derived from eight organs (e.g., callus, seedling shoot, seedling root, leaf at the tillering stage, leaf at the flowering stage, booting panicle, flowering panicle, and filling panicle) of indica rice identified 7232 novel transcription units (Zhang et al. 2010). Likewise, other cereal crops like sorghum seedling transcriptome using single-molecule long reads reveal 27,860 FLNC transcripts, of which 11,342 (40.7%) were novel and over 2100 novel genes corresponded to ~ 11,000 novel spliced isoforms (Abdel-Ghany et al. 2016). It is evident that chimeric fusion events are common in plants and further inflate the complexity of the transcriptome (Wang et al. 2016; Zhang et al. 2010). Our data reveals 6.8% (2285) of the total spliced isoforms are Fusion isoforms which is higher than found with RNA-Seq data (234 rice fusion events). These transcripts may arise from trans-splicing (Zhang et al. 2010).

The isoforms structure of 19 starch synthesis-related genes was investigated to illustrate the complexity of the transcriptome involved in a single metabolic process. This revealed a total of 276 isoforms related to starch synthesis genes of which 94 isoforms were novel. A large number of splicing isoforms was found with 51 for *SSIIIa* (51) followed by 45 for starch branching enzyme (*BEIIa*), 28 for *GBSSI*, and *BEIIb* each. Intron retention and at least one novel splice site primarily result in novel isoforms. The rice genome encodes 27 starch synthesis related genes which belong to 6 classes of enzymes: AGPase subunits with 6 isoforms, starch synthase (SS) with 10 isoforms, starch branching enzyme (SBE) with 3 isoforms, and starch debranching enzyme (DBE) with 4 isoforms, starch phosphorylase (PHOL) with 2 isoforms and disproportionating enzyme (DPE) with 2 isoforms (Ohdan et al. 2005). However, many of the starch synthesis-related spliced isoforms reported here have not been identified in earlier transcriptome studies.

Alternative splicing (AS) has been more comprehensively explored in animals than in plants (Wang and Brendle 2006). Alternative splicing (AS) is an effective system for genomes to make a great diversity and complexity of transcripts. The identification of the many transcripts resulting from AS of rice genes will support increased understanding of the processes of post-transcriptional regulation of gene expression and function (Lu et al. 2010). The Iso-Seq data identified a large number of splice junctions (73,659) of which 82.65% were known canonical with GT-AG, GC-AG, and AT-AC intron boundaries, and 17.32% novel splice junctions with canonical and non-canonical intron boundaries were identified. Previous estimates from RNA-Seq data suggested that over 99% of intron boundaries in the rice transcriptome were GT-AG (Lu et al. 2010; Zhang et al. 2019). This study reveals many more splicing junctions compared to the previous studies in Nipponbare reporting 68,441 unique GT–AG splicing junctions (Lu et al. 2010), 64,513 splicing junctions (Zhang et al. 2019), and the genome annotation databases or public transcriptomic data such as MSU_orsa1r7, Rice Annotation Project Database (RAP-DB), National Center for Biotechnology Information (NCBI) (Zhang et al. 2019). The number of AS events (6461) in this study was higher than that found using a RSII system (1889) (Zhang et al. 2019). Intron retention appears to be the dominant alternative splicing event in generating splicing isoforms and exon skipping is the least prevalent alternative event. This agrees with the previous reports in plants including rice (Wang and Brendel 2006; Zhang et al. 2010, 2019), Arabidopsis (Wang and Brendel 2006), maize (Wang et al. 2016), and sorghum (Abdel-Ghany et al. 2016). In contrast, exon skipping is the most prevalent mechanism in animals (Sultan et al. 2008; Wang et al. 2008).

The isoforms did include many that were probably produced by degradation resulting from nuclease action on the 5′ end of transcripts. This is illustrated in the transcripts for *GBSSI* (Fig. 3b). The removal of the start codon and upstream sequences associated with ribosomal binding to the transcript would result in the transcript no longer being translated into protein. The smaller number of isoforms found for the novel genes may be due to these being genes expressed at low levels. This could explain they are being omitted in annotations and to the detection of so few variants.

Most of the Iso-Seq transcripts were annotated against the Viridiplantae database and had homolog to the protein database. The unannotated sequences were subjected to RNA family detection. Only three sequences had homology to those in the RNA databases. The remaining unannotated sequences have been shown to be potential non-coding RNAs and are predicted sncRNA and lncRNA based on their length. Predicted ncRNAs, including 03 lncRNAs, seem to be novel as these have not been reported in any of the public databases. ncRNAs do not translate into proteins but can regulate gene expression. The identification of these ncRNA has the potential to help explain their regulatory function in the cellular process of an organism (Zhang et al. 2019). Next-generation high-throughput RNA sequencing technologies have the resolution to capture many novel transcripts exhibiting ncRNA. For instance, 471 transcripts were predicted to be long non-coding RNAs (lncRNAs) in a rice seedling transcriptome (Zhang et al. 2019). Transcription factors (TFs) are essential for turning on and off genes of many morphological and biological processes and functions in a coordinated fashion (Gao et al. 2021). Our data reveal a slightly greater number of transcription factors belonging to CAMTA, HB-other, ARF, C3H, TALE, GATA, Co-like, and SBP TF family compared to the number of TFs previously reported in the annotated genome of rice (MSU_osa1r7) and available in the plant transcript factor database (PlantTFDB version 5.0) (Jin et al. 2017). These newly identified TFs may contribute to understanding the mechanism of transcription control. KEGG pathway analysis reveals the largest number of transcript isoforms are involved in the starch and sucrose metabolism pathway.

One of the most important findings of this study is the characterization of a substantial number of novel isoforms from different alternative splicing events and novel genes. Not all the splice variants will have a major role in the cellular and biological process. For instance, ~ 45% of the isoforms detected in maize and sorghum have been shown to decay before transportation to the cell (Wang et al. 2018). However, many isoforms from novel genes suggest the presence of coding regions that have not yet been discovered.

## Conclusion

The study reveals a substantial number of high-quality full-length transcripts in the Nipponbare rice transcriptome. Transcriptome analysis also reveals a number of novel genes that were not reported in the reference genome. Around 56% of the isoforms (FSM and ISM) were annotated to the reference transcripts while the other isoforms were novel. The novel genes and isoforms are worth further investigation. This study also reveals a large diversity of splicing junctions including known (i.e., already reported in the reference transcript) as well as novel splice junctions. Intron retention was the prominent alternative splicing event. Novel isoforms were abundant, mainly due to the presence of intron retention and at least one novel splice site in the isoform structure. Many of the novel isoforms showed coding potential inferring that they might be functional in cellular and metabolic processes. The analysis predicts a number of non-coding RNAs from the data as well as a number of additional transcription factors. This information will be helpful in future rice transcriptome investigations as well as improving the current rice reference genome.

## Supplementary Information

**Additional file 1. Table S1**: Alternative splicing events of 33,504 annotated HQ transcripts.

**Additional file 2. Table S2**: Summary of the coding probability of non-coding RNA from the coding potential calculator 2 (CPC2) web server (Kang et al. 2017). Protein length < 200 amino acid (aa) were regarded as small non-coding RNAs (sncRNA) while those with protein length ≥ 200 aa were regarded as lncRNA.

**Additional file 3. Fig S1**: Length distribution of all FLNC transcripts. Two peaks corresponding to two library bin sizes of > 4kb and < 4kb.

**Additional file 4. Fig S2**: Comparison of plant transcription factors of O. sativa ssp. japonica var. Nipponbare obtained from Iso-Seq transcripts from PacBio Sequel platform and MSU Rice Genome Annotation Project Release 7, MSU (v.7) obtained from the plant transcription factor database (PlantTFDB version 5.0) (Jin et al. 2017).

**Additional file 5. Fig S3**: Top ten Kyoto Encyclopedia of Genes and Genomics (KEGG) pathways represented by the highest number of transcripts linked to the pathway categories of genetic information processing, cellular process, environmental information processing, organismal systems, and metabolism in all the aligned transcript isoforms.

## Data Availability

Datasets used and described in this study are made available for non-commercial research purposes. The data are openly available in Sequence Read Archive (SRA) at https://www.ncbi.nlm, under the BioProject number PRJNA813759.

## Abbreviations

AS: Alternative splicing
FLNC: Full-length non-chimeric reads
Pre-mRNA: Precursor mRNA
mRNA: Messenger RNA
RT-PCR: Reverse transcription PCR
SAGE: Serial analysis of gene expression
PATTY: PCR aided transcript titration assay
RNA-Seq: RNA sequencing
SMRT: Single molecule real-time
PacBio: Pacific Biosciences
Iso-Seq: Isoform-sequencing
APA: Alternative polyadenylation
MTP: Minimum tiling path
cDNA: Complementary DNA
Poly-A: Polyadenylic acid
SMARTer: Switching Mechanism at 5′ End of RNA Template
UTRs: Untranslated regions
RIN: RNA integrity number
CCS: Circular consensus sequence
FL: Full-length
NFL: Non-full length
ROIs: Read of inserts
ICE: Iterative clustering and error correction
HQ: High-quality
LQ: Low-quality
SQANTI: Structural and Quality Annotation of Novel Transcripts Isoforms
ORF: Open reading frame
*AGPL2*: *ADP-glucose pyrophosphorylase large subunit 2*
*GBSSI*: *Granule bound starch synthase I*
*GBSSII*: *Granule bound starch synthase II*
*SSI*: *Soluble starch synthase I*
*SSIIa*: *Soluble starch synthase IIa*
*SSIIb*: *Soluble starch synthase IIb*
*SSIIc*: *Soluble starch synthase IIc*
*SSIIIa*: *Soluble starch synthase III-2*
*SSIIIb*: *Soluble starch synthase III-1*
*SSIVa*: *Soluble starch synthase IV-1*
*SSIVb*: *Soluble starch synthase IV-2*
*BEI*: *Starch branching enzyme I*
*BEIIa*: *Starch branching enzyme IIa*
*BEIIb*: *Starch branching enzyme IIb*
*ISA1*: *Isoamylase 1*
*ISA2*: *Isoamylase 2*
*PUL*: *Pullulanase*
*GPT1*: *Glucose-6-phosphate translocator*
*PHOL*: *Starch phosphorylase*
*DPE*: Disproportionating enzyme
IGV: Integrated genomics viewer
AStalavista: Alternative splicing transcriptional landscape visualization tool
FSM: Full splice match
ISM: Incomplete splice match
NIC: Novel In Catalog
NNC: Novel Not in Catalog
BLAST: Basic Local Alignment Search Tool
GO: Gene ontology
lncRNA: Long non-coding RNA
ncRNA: Non-coding RNA
sncRNA: Small non-coding RNA
TF: Transcription factor
KEGG: Kyoto Encyclopedia of Genes and Genomics
RISC complex: RNA-induced silencing complex
RNA: Ribonucleic acid
miRNA: Micro-RNA
tRNA: Transfer RNA
DPA: Days postanthesis
EMBL-EBI: EMBL’s European Bioinformatics Institute
NCBI: National Center for Biotechnology Information
