# Supplementary material for: The Long Read Transcriptome of Rice (Oryza sativa ssp. japonica var. Nipponbare) Reveals Novel Transcripts
Source: Rice (N Y). 2022 Jun 11;15:29. doi: 10.1186/s12284-022-00577-1 (PMC9188635; doi:10.1186/s12284-022-00577-1)

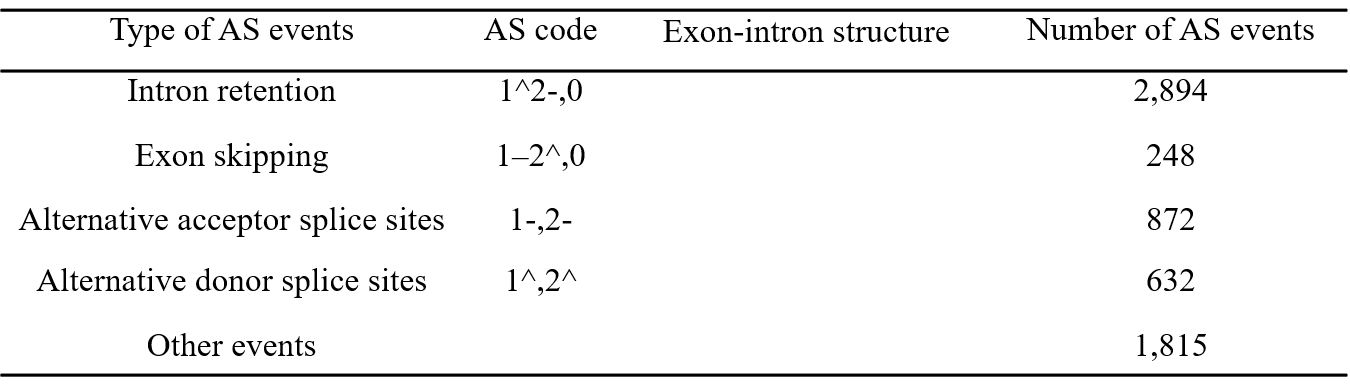
Supplementary Table 1: Alternative splicing events of 33,504 annotated HQ transcripts using AStalavista version 5 software.


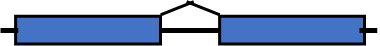

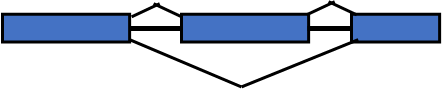

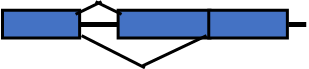

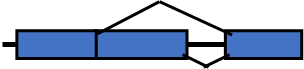

Supplement: Supplementary file 1 — Additional file 1. Table S1: Alternative splicing events of 33,504 annotated HQ transcripts. [file 12284_2022_577_MOESM1_ESM.docx]
