# Supplementary material for: The Long Read Transcriptome of Rice (Oryza sativa ssp. japonica var. Nipponbare) Reveals Novel Transcripts
Source: Rice (N Y). 2022 Jun 11;15:29. doi: 10.1186/s12284-022-00577-1 (PMC9188635; doi:10.1186/s12284-022-00577-1)
Supplement: Supplementary file 2 — Additional file 2. Table S2: Summary of the coding probability of non-coding RNA from the coding potential calculator 2 (CPC2) web server (Kang et al. 2017). Protein length < 200 amino acid (aa) were regarded as small non-coding RNAs (sncRNA) while those with protein length ≥ 200 aa were regarded as lncRNA. [file 12284_2022_577_MOESM2_ESM.docx]

Supplementary Table 2: Summary of the coding probability of non-coding RNA from CPC2 web server.

| **Transcript ID** | **Label** | **Coding probability** | **Peptide length(aa)** | **Fickett score** | **Isoelectric point** | **ORF integrity** |
| --- | --- | --- | --- | --- | --- | --- |
| transcript/16893 | noncoding | 0.450283 | 216 | 0.31464 | 12.3637085 | complete |
| transcript/16431 | noncoding | 0.351993 | 211 | 0.2767 | 11.64447021 | complete |
| transcript/31120 | noncoding | 0.267829 | 200 | 0.27942 | 12.00408936 | complete |
| transcript/9683 | noncoding | 0.266061 | 153 | 0.32377 | 11.93414307 | complete |
| transcript/16585 | noncoding | 0.146834 | 151 | 0.25433 | 11.60980225 | complete |
| transcript/31872 | noncoding | 0.458349 | 147 | 0.35869 | 11.53033447 | complete |
| transcript/30571 | noncoding | 0.165335 | 142 | 0.33074 | 9.35369873 | incomplete |
| transcript/32705 | noncoding | 0.147705 | 137 | 0.34167 | 11.86029053 | incomplete |
| transcript/30761 | noncoding | 0.248592 | 134 | 0.37037 | 12.47772217 | complete |
| transcript/21024 | noncoding | 0.153742 | 132 | 0.27712 | 10.74127197 | complete |
| transcript/15175 | noncoding | 0.190672 | 131 | 0.28247 | 9.355407715 | complete |
| transcript/30730 | noncoding | 0.157772 | 126 | 0.33455 | 12.1416626 | complete |
| transcript/18713 | noncoding | 0.28906 | 111 | 0.32546 | 6.261535645 | complete |
| transcript/32473 | noncoding | 0.376409 | 105 | 0.40411 | 9.623840332 | complete |
| transcript/32716 | noncoding | 0.400854 | 102 | 0.41788 | 9.440124512 | complete |
| transcript/32564 | noncoding | 0.490857 | 101 | 0.38445 | 5.144714355 | complete |
| transcript/31836 | noncoding | 0.471696 | 96 | 0.4817 | 6.29107666 | complete |
| transcript/31962 | noncoding | 0.0692898 | 96 | 0.31922 | 12.1519165 | complete |
| transcript/12969 | noncoding | 0.105883 | 95 | 0.32741 | 10.49505615 | complete |
| transcript/32067 | noncoding | 0.0603134 | 94 | 0.28646 | 12.12506104 | complete |
| transcript/31888 | noncoding | 0.395321 | 93 | 0.45339 | 6.70135498 | complete |
| transcript/32301 | noncoding | 0.294851 | 91 | 0.44957 | 12.41778564 | complete |
| transcript/32531 | noncoding | 0.168495 | 91 | 0.40106 | 11.88336182 | complete |
| transcript/31401 | noncoding | 0.402618 | 89 | 0.3384 | 3.679016113 | complete |
| transcript/32123 | noncoding | 0.0491673 | 83 | 0.27975 | 12.30108643 | complete |
| transcript/32397 | noncoding | 0.34014 | 81 | 0.40386 | 4.58404541 | complete |
| transcript/32413 | noncoding | 0.359445 | 81 | 0.41137 | 4.58404541 | complete |
| transcript/32471 | noncoding | 0.34014 | 81 | 0.40386 | 4.58404541 | complete |
| transcript/31550 | noncoding | 0.051669 | 80 | 0.32459 | 12.06817627 | complete |
| transcript/33001 | noncoding | 0.147163 | 80 | 0.40994 | 8.880065918 | complete |
| transcript/22426 | noncoding | 0.047374 | 79 | 0.30357 | 8.368591309 | complete |
| transcript/17148 | noncoding | 0.0533543 | 78 | 0.32524 | 8.798156738 | complete |
| transcript/32527 | noncoding | 0.215283 | 77 | 0.42703 | 5.589782715 | complete |
| transcript/32756 | noncoding | 0.0790692 | 77 | 0.36641 | 11.48309326 | complete |
| transcript/31096 | noncoding | 0.207378 | 76 | 0.32907 | 3.807678223 | complete |
| transcript/33060 | noncoding | 0.0452847 | 76 | 0.31758 | 8.165222168 | complete |
| transcript/33076 | noncoding | 0.0286626 | 75 | 0.3673 | 4.641662598 | incomplete |
| transcript/16257 | noncoding | 0.06344 | 72 | 0.37126 | 11.82757568 | complete |
| transcript/2451 | noncoding | 0.0605571 | 71 | 0.25037 | 6.889465332 | complete |
| transcript/2517 | noncoding | 0.0424573 | 71 | 0.27907 | 6.889465332 | complete |
| transcript/2526 | noncoding | 0.0424573 | 71 | 0.27907 | 6.889465332 | complete |
| transcript/2553 | noncoding | 0.0424573 | 71 | 0.27907 | 6.889465332 | complete |
| transcript/32313 | noncoding | 0.21147 | 71 | 0.44831 | 10.57220459 | complete |
| transcript/32805 | noncoding | 0.0555938 | 71 | 0.35476 | 6.515563965 | complete |
| transcript/32106 | noncoding | 0.0792389 | 70 | 0.40415 | 8.273132324 | complete |
| transcript/32252 | noncoding | 0.132132 | 70 | 0.46161 | 6.960632324 | complete |
| transcript/32476 | noncoding | 0.126258 | 70 | 0.45333 | 6.960632324 | complete |
| transcript/32662 | noncoding | 0.0847612 | 70 | 0.40665 | 6.828796387 | complete |
| transcript/33041 | noncoding | 0.0532731 | 69 | 0.37497 | 7.694519043 | complete |
| transcript/33128 | noncoding | 0.0363427 | 69 | 0.32444 | 8.557678223 | complete |
| transcript/31794 | noncoding | 0.0439124 | 68 | 0.31293 | 11.19024658 | complete |
| transcript/33102 | noncoding | 0.0792041 | 68 | 0.4002 | 9.358215332 | complete |
| transcript/32807 | noncoding | 0.100511 | 67 | 0.44293 | 7.828674316 | complete |
| transcript/32865 | noncoding | 0.054142 | 67 | 0.36451 | 11.61627197 | complete |
| transcript/33213 | noncoding | 0.0903419 | 67 | 0.39017 | 5.496154785 | complete |
| transcript/31933 | noncoding | 0.0407063 | 66 | 0.31283 | 11.54656982 | complete |
| transcript/32079 | noncoding | 0.0461547 | 66 | 0.34421 | 11.54656982 | complete |
| transcript/32166 | noncoding | 0.119072 | 66 | 0.43969 | 5.753234863 | complete |
| transcript/32328 | noncoding | 0.0460568 | 66 | 0.34385 | 11.54656982 | complete |
| transcript/32540 | noncoding | 0.10488 | 66 | 0.43957 | 12.32281494 | complete |
| transcript/32637 | noncoding | 0.159826 | 66 | 0.45908 | 12.32281494 | complete |
| transcript/32702 | noncoding | 0.147163 | 66 | 0.45547 | 12.32281494 | complete |
| transcript/33040 | noncoding | 0.118114 | 66 | 0.4408 | 12.1461792 | complete |
| transcript/33120 | noncoding | 0.0876912 | 66 | 0.40574 | 11.21929932 | complete |
| transcript/31856 | noncoding | 0.130192 | 64 | 0.35017 | 3.764221191 | complete |
| transcript/32488 | noncoding | 0.0814248 | 64 | 0.41075 | 9.875183105 | complete |
| transcript/32601 | noncoding | 0.207601 | 64 | 0.41511 | 4.155456543 | complete |
| transcript/32737 | noncoding | 0.182714 | 64 | 0.46468 | 9.875183105 | complete |
| transcript/33035 | noncoding | 0.0878991 | 64 | 0.41227 | 10.30218506 | complete |
| transcript/3634 | noncoding | 0.0484426 | 64 | 0.27359 | 5.965881348 | complete |
| transcript/32153 | noncoding | 0.0605712 | 63 | 0.40971 | 8.518981934 | complete |
| transcript/32311 | noncoding | 0.059434 | 63 | 0.40806 | 8.518981934 | complete |
| transcript/32733 | noncoding | 0.0509301 | 63 | 0.38947 | 8.860046387 | complete |
| transcript/32860 | noncoding | 0.097574 | 63 | 0.46408 | 7.793151855 | complete |
| transcript/33192 | noncoding | 0.0688203 | 63 | 0.41577 | 8.860046387 | complete |
| transcript/32175 | noncoding | 0.0600557 | 62 | 0.42037 | 7.858703613 | complete |
| transcript/32933 | noncoding | 0.0216243 | 62 | 0.38283 | 4.104797363 | incomplete |
| transcript/11064 | noncoding | 0.0368732 | 61 | 0.31981 | 11.41766357 | complete |
| transcript/33383 | noncoding | 0.0435637 | 61 | 0.41675 | 8.016052246 | incomplete |
| transcript/32568 | noncoding | 0.171195 | 59 | 0.46639 | 10.43548584 | complete |
| transcript/33346 | noncoding | 0.0532675 | 59 | 0.38319 | 5.464660645 | complete |
| transcript/32472 | noncoding | 0.0386741 | 58 | 0.39881 | 7.976989746 | complete |
| transcript/32585 | noncoding | 0.0465674 | 58 | 0.41639 | 7.976989746 | complete |
| transcript/32644 | noncoding | 0.0633808 | 58 | 0.45014 | 7.633972168 | complete |
| transcript/32757 | noncoding | 0.0690717 | 58 | 0.36823 | 4.538757324 | complete |
| transcript/32856 | noncoding | 0.0768697 | 58 | 0.47575 | 7.633972168 | complete |
| transcript/32911 | noncoding | 0.0522139 | 58 | 0.42343 | 6.568054199 | complete |
| transcript/12068 | noncoding | 0.0228789 | 57 | 0.30525 | 6.487121582 | complete |
| transcript/32745 | noncoding | 0.0884714 | 57 | 0.4334 | 10.45672607 | complete |
| transcript/33036 | noncoding | 0.0253443 | 57 | 0.36087 | 7.649841309 | complete |
| transcript/19657 | noncoding | 0.0358797 | 56 | 0.33535 | 5.107727051 | complete |
| transcript/20163 | noncoding | 0.0394673 | 56 | 0.3524 | 5.107727051 | complete |
| transcript/24461 | noncoding | 0.053371 | 56 | 0.3843 | 5.107727051 | complete |
| transcript/33249 | noncoding | 0.0214054 | 56 | 0.33195 | 6.679016113 | complete |
| transcript/31994 | noncoding | 0.0217563 | 55 | 0.33872 | 8.517272949 | complete |
| transcript/32707 | noncoding | 0.027337 | 55 | 0.3819 | 7.652893066 | complete |
| transcript/29617 | noncoding | 0.0215808 | 54 | 0.2792 | 9.033752441 | complete |
| transcript/32814 | noncoding | 0.0764703 | 54 | 0.41105 | 9.389831543 | incomplete |
| transcript/32878 | noncoding | 0.0615434 | 54 | 0.39858 | 8.750671387 | incomplete |
| transcript/33093 | noncoding | 0.0787453 | 54 | 0.41647 | 9.508728027 | incomplete |
| transcript/33177 | noncoding | 0.0680089 | 54 | 0.38542 | 8.750671387 | incomplete |
| transcript/33094 | noncoding | 0.0489298 | 53 | 0.37126 | 4.676940918 | complete |
| transcript/33135 | noncoding | 0.0350566 | 53 | 0.41729 | 7.842590332 | complete |
| transcript/31911 | noncoding | 0.107648 | 53 | 0.34322 | 8.930603027 | incomplete |
| transcript/33272 | noncoding | 0.081401 | 53 | 0.37542 | 8.987976074 | incomplete |
| transcript/26601 | noncoding | 0.0236602 | 52 | 0.33017 | 9.698425293 | complete |
| transcript/32235 | noncoding | 0.0382215 | 52 | 0.40577 | 12.20513916 | complete |
| transcript/33254 | noncoding | 0.027219 | 52 | 0.38423 | 8.684265137 | complete |
| transcript/32882 | noncoding | 0.0398184 | 52 | 0.35098 | 6.50213623 | incomplete |
| transcript/32800 | noncoding | 0.0355225 | 51 | 0.3978 | 12.09686279 | complete |
| transcript/32902 | noncoding | 0.0279152 | 51 | 0.37654 | 5.727966309 | complete |
| transcript/32985 | noncoding | 0.0292639 | 51 | 0.40142 | 8.47479248 | complete |
| transcript/33000 | noncoding | 0.0228219 | 51 | 0.38601 | 7.629455566 | complete |
| transcript/33052 | noncoding | 0.0280957 | 51 | 0.3735 | 5.635437012 | complete |
| transcript/33283 | noncoding | 0.0202644 | 51 | 0.36303 | 6.491149902 | complete |
| transcript/32567 | noncoding | 0.0212315 | 51 | 0.34505 | 4.36895752 | incomplete |
| transcript/11799 | noncoding | 0.0206167 | 50 | 0.32072 | 9.554626465 | complete |
| transcript/2628 | noncoding | 0.0343709 | 50 | 0.25049 | 10.29498291 | complete |
| transcript/32906 | noncoding | 0.0285044 | 50 | 0.35556 | 11.7855835 | complete |
| transcript/33201 | noncoding | 0.0300552 | 50 | 0.37194 | 10.04425049 | complete |
| transcript/7312 | noncoding | 0.0281761 | 50 | 0.2671 | 10.29498291 | complete |
| transcript/31418 | noncoding | 0.055463 | 50 | 0.44645 | 9.066467285 | incomplete |
| transcript/33167 | noncoding | 0.0857541 | 50 | 0.39139 | 9.446105957 | incomplete |
| transcript/33547 | noncoding | 0.0238669 | 50 | 0.43454 | 6.692199707 | incomplete |
| transcript/32871 | noncoding | 0.111904 | 49 | 0.44068 | 4.020568848 | complete |
| transcript/33086 | noncoding | 0.0166889 | 49 | 0.35817 | 7.761291504 | complete |
| transcript/33245 | noncoding | 0.0350245 | 49 | 0.28445 | 5.002258301 | complete |
| transcript/33395 | noncoding | 0.073145 | 49 | 0.38902 | 9.066467285 | incomplete |
| transcript/32615 | noncoding | 0.0281847 | 48 | 0.36935 | 11.83233643 | complete |
| transcript/32779 | noncoding | 0.0994573 | 48 | 0.45708 | 4.078918457 | complete |
| transcript/32862 | noncoding | 0.101591 | 48 | 0.44906 | 4.078918457 | complete |
| transcript/32960 | noncoding | 0.0535677 | 48 | 0.45538 | 9.010192871 | complete |
| transcript/33434 | noncoding | 0.0492552 | 48 | 0.42628 | 10.13580322 | complete |
| transcript/33271 | noncoding | 0.0211305 | 48 | 0.40639 | 5.938293457 | incomplete |
| transcript/32695 | noncoding | 0.0150115 | 47 | 0.31105 | 8.737487793 | complete |
| transcript/32870 | noncoding | 0.0269163 | 47 | 0.40834 | 8.752624512 | complete |
| transcript/33023 | noncoding | 0.0172412 | 47 | 0.375 | 6.681945801 | complete |
| transcript/33164 | noncoding | 0.0360543 | 47 | 0.42466 | 5.577331543 | complete |
| transcript/33400 | noncoding | 0.0653922 | 47 | 0.41557 | 9.236633301 | incomplete |
| transcript/33440 | noncoding | 0.0540628 | 47 | 0.35818 | 7.680725098 | incomplete |
| transcript/33569 | noncoding | 0.0634548 | 47 | 0.36899 | 8.358825684 | incomplete |
| transcript/32468 | noncoding | 0.0325708 | 46 | 0.4153 | 9.414611816 | complete |
| transcript/32514 | noncoding | 0.0268934 | 46 | 0.35765 | 11.13751221 | complete |
| transcript/32931 | noncoding | 0.0289592 | 46 | 0.42677 | 6.058410645 | complete |
| transcript/33113 | noncoding | 0.0410414 | 46 | 0.45303 | 5.410461426 | complete |
| transcript/33160 | noncoding | 0.0255914 | 46 | 0.40738 | 8.803771973 | complete |
| transcript/33118 | noncoding | 0.0643368 | 46 | 0.43487 | 9.389709473 | incomplete |
| transcript/33207 | noncoding | 0.0575933 | 46 | 0.41506 | 8.987365723 | incomplete |
| transcript/33263 | noncoding | 0.0270687 | 46 | 0.39708 | 6.889099121 | incomplete |
| transcript/32872 | noncoding | 0.0263995 | 45 | 0.42678 | 8.295837402 | complete |
| transcript/31405 | noncoding | 0.0168775 | 45 | 0.36954 | 4.36895752 | incomplete |
| transcript/33127 | noncoding | 0.0625753 | 45 | 0.37498 | 8.503967285 | incomplete |
| transcript/33298 | noncoding | 0.0219155 | 45 | 0.46458 | 6.692687988 | incomplete |
| transcript/33344 | noncoding | 0.0308266 | 45 | 0.43216 | 7.789978027 | incomplete |
| transcript/33506 | noncoding | 0.0219185 | 45 | 0.46388 | 6.692687988 | incomplete |
| transcript/32907 | noncoding | 0.0362225 | 44 | 0.41406 | 5.002258301 | complete |
| transcript/33248 | noncoding | 0.0261015 | 44 | 0.42033 | 8.769836426 | complete |
| transcript/33338 | noncoding | 0.0196065 | 44 | 0.34183 | 5.002258301 | complete |
| transcript/33342 | noncoding | 0.0158343 | 44 | 0.38461 | 7.907409668 | complete |
| transcript/33447 | noncoding | 0.0203529 | 44 | 0.34992 | 5.002258301 | complete |
| transcript/32892 | noncoding | 0.0230619 | 44 | 0.31771 | 3.998840332 | incomplete |
| transcript/33054 | noncoding | 0.019687 | 44 | 0.43892 | 5.682312012 | incomplete |
| transcript/33107 | noncoding | 0.0748747 | 44 | 0.39125 | 9.236633301 | incomplete |
| transcript/32336 | noncoding | 0.0143546 | 43 | 0.36653 | 8.402526855 | complete |
| transcript/33114 | noncoding | 0.0109873 | 43 | 0.32081 | 7.81854248 | complete |
| transcript/33124 | noncoding | 0.0446964 | 43 | 0.41478 | 4.55657959 | complete |
| transcript/33206 | noncoding | 0.0634338 | 43 | 0.46919 | 9.624694824 | complete |
| transcript/33288 | noncoding | 0.0244291 | 43 | 0.44015 | 7.799377441 | complete |
| transcript/33368 | noncoding | 0.0341577 | 43 | 0.46063 | 5.36151123 | complete |
| transcript/33480 | noncoding | 0.0274722 | 43 | 0.41154 | 5.376281738 | complete |
| transcript/33156 | noncoding | 0.0306394 | 43 | 0.48123 | 4.307678223 | incomplete |
| transcript/33260 | noncoding | 0.0638888 | 43 | 0.29198 | 5.409606934 | incomplete |
| transcript/33437 | noncoding | 0.0207163 | 43 | 0.45236 | 6.520202637 | incomplete |
| transcript/33489 | noncoding | 0.119705 | 43 | 0.24611 | 4.36895752 | incomplete |
| transcript/32138 | noncoding | 0.0424327 | 42 | 0.37883 | 3.946105957 | complete |
| transcript/32811 | noncoding | 0.011059 | 42 | 0.34628 | 7.753601074 | complete |
| transcript/32845 | noncoding | 0.0307097 | 42 | 0.46749 | 7.978942871 | complete |
| transcript/33055 | noncoding | 0.025109 | 42 | 0.3753 | 10.83416748 | complete |
| transcript/32997 | noncoding | 0.160164 | 42 | 0.26678 | 7.708068848 | incomplete |
| transcript/33045 | noncoding | 0.0240261 | 42 | 0.32369 | 4.36895752 | incomplete |
| transcript/33258 | noncoding | 0.175273 | 42 | 0.36922 | 11.45318604 | incomplete |
| transcript/33280 | noncoding | 0.0465919 | 41 | 0.41468 | 4.300720215 | complete |
| transcript/33397 | noncoding | 0.0147283 | 41 | 0.38467 | 8.404846191 | complete |
| transcript/33411 | noncoding | 0.0340107 | 41 | 0.41424 | 11.47479248 | complete |
| transcript/33173 | noncoding | 0.0694102 | 41 | 0.38289 | 8.96661377 | incomplete |
| transcript/33446 | noncoding | 0.0192644 | 41 | 0.44654 | 5.463439941 | incomplete |
| transcript/33599 | noncoding | 0.0745362 | 41 | 0.37533 | 8.96661377 | incomplete |
| transcript/32722 | noncoding | 0.0186104 | 40 | 0.3921 | 5.425842285 | complete |
| transcript/32755 | noncoding | 0.022177 | 40 | 0.41145 | 5.425842285 | complete |
| transcript/32883 | noncoding | 0.0223757 | 40 | 0.45474 | 6.108459473 | complete |
| transcript/32954 | noncoding | 0.0220744 | 40 | 0.41091 | 5.425842285 | complete |
| transcript/33418 | noncoding | 0.0121583 | 40 | 0.37841 | 6.497131348 | complete |
| transcript/33233 | noncoding | 0.0933253 | 40 | 0.36409 | 9.245300293 | incomplete |
| transcript/33409 | noncoding | 0.050246 | 40 | 0.36096 | 7.728942871 | incomplete |
| transcript/32832 | noncoding | 0.0386459 | 39 | 0.47061 | 8.937561035 | complete |
| transcript/32849 | noncoding | 0.0221162 | 39 | 0.45328 | 7.894836426 | complete |
| transcript/32978 | noncoding | 0.0145358 | 39 | 0.38993 | 5.873718262 | complete |
| transcript/33020 | noncoding | 0.016049 | 39 | 0.40084 | 8.658752441 | complete |
| transcript/33187 | noncoding | 0.025698 | 39 | 0.44392 | 8.751037598 | complete |
| transcript/33354 | noncoding | 0.0252674 | 39 | 0.4417 | 5.425231934 | complete |
| transcript/33387 | noncoding | 0.0200043 | 39 | 0.43626 | 8.160705566 | complete |
| transcript/33323 | noncoding | 0.0799361 | 39 | 0.35312 | 8.610534668 | incomplete |
| transcript/32715 | noncoding | 0.0140213 | 38 | 0.40887 | 7.953430176 | complete |
| transcript/32867 | noncoding | 0.0289877 | 38 | 0.41767 | 11.87542725 | complete |
| transcript/32992 | noncoding | 0.0109651 | 38 | 0.37712 | 7.953430176 | complete |
| transcript/33269 | noncoding | 0.0272409 | 38 | 0.4232 | 9.89642334 | complete |
| transcript/33311 | noncoding | 0.0184194 | 38 | 0.37082 | 4.780456543 | complete |
| transcript/33481 | noncoding | 0.0149877 | 38 | 0.42228 | 6.674133301 | complete |
| transcript/33565 | noncoding | 0.0209581 | 38 | 0.44268 | 5.74822998 | complete |
| transcript/32692 | noncoding | 0.0128839 | 38 | 0.3635 | 3.665710449 | incomplete |
| transcript/32706 | noncoding | 0.0871646 | 38 | 0.40112 | 9.80670166 | incomplete |
| transcript/33158 | noncoding | 0.0632571 | 38 | 0.34505 | 7.821716309 | incomplete |
| transcript/33426 | noncoding | 0.137705 | 38 | 0.2852 | 7.859069824 | incomplete |
| transcript/33532 | noncoding | 0.0292104 | 38 | 0.44095 | 7.957702637 | incomplete |
| transcript/32172 | noncoding | 0.0125416 | 37 | 0.34776 | 9.300842285 | complete |
| transcript/32598 | noncoding | 0.00932397 | 37 | 0.29183 | 7.799377441 | complete |
| transcript/32761 | noncoding | 0.0200814 | 37 | 0.43884 | 8.508850098 | complete |
| transcript/33111 | noncoding | 0.0134277 | 37 | 0.364 | 9.300231934 | complete |
| transcript/33126 | noncoding | 0.0149303 | 37 | 0.42685 | 6.491271973 | complete |
| transcript/33143 | noncoding | 0.054219 | 37 | 0.44328 | 3.971252441 | complete |
| transcript/33215 | noncoding | 0.00871265 | 37 | 0.34841 | 7.745788574 | complete |
| transcript/33227 | noncoding | 0.00925648 | 37 | 0.36312 | 6.491271973 | complete |
| transcript/33257 | noncoding | 0.0660019 | 37 | 0.47669 | 10.16595459 | complete |
| transcript/33371 | noncoding | 0.0115424 | 37 | 0.37573 | 8.504455566 | complete |
| transcript/33579 | noncoding | 0.0112614 | 37 | 0.39124 | 6.491271973 | complete |
| transcript/32929 | noncoding | 0.021414 | 37 | 0.39575 | 6.486877441 | incomplete |
| transcript/33427 | noncoding | 0.0177973 | 37 | 0.43736 | 5.108093262 | incomplete |
| transcript/32949 | noncoding | 0.0130018 | 36 | 0.41698 | 6.501159668 | complete |
| transcript/32970 | noncoding | 0.0268916 | 36 | 0.45247 | 9.057556152 | complete |
| transcript/32994 | noncoding | 0.00945863 | 36 | 0.29278 | 6.50604248 | complete |
| transcript/33097 | noncoding | 0.0134601 | 36 | 0.38415 | 9.057556152 | complete |
| transcript/33244 | noncoding | 0.0142835 | 36 | 0.29189 | 5.292419434 | complete |
| transcript/33347 | noncoding | 0.0079219 | 36 | 0.33217 | 7.675354004 | complete |
| transcript/33468 | noncoding | 0.0188526 | 36 | 0.42263 | 9.057556152 | complete |
| transcript/33059 | noncoding | 0.0169239 | 36 | 0.43069 | 5.702209473 | incomplete |
| transcript/33380 | noncoding | 0.162229 | 36 | 0.41655 | 11.8348999 | incomplete |
| transcript/33576 | noncoding | 0.0547633 | 36 | 0.40671 | 8.96661377 | incomplete |
| transcript/32923 | noncoding | 0.0332819 | 35 | 0.45235 | 9.762878418 | complete |
| transcript/33019 | noncoding | 0.0179734 | 35 | 0.43222 | 8.75177002 | complete |
| transcript/33068 | noncoding | 0.0140291 | 35 | 0.35927 | 4.778259277 | complete |
| transcript/33129 | noncoding | 0.00977891 | 35 | 0.38833 | 6.486877441 | complete |
| transcript/33149 | noncoding | 0.00976866 | 35 | 0.31147 | 8.850402832 | complete |
| transcript/33151 | noncoding | 0.0103434 | 35 | 0.29404 | 8.840637207 | complete |
| transcript/33287 | noncoding | 0.0139461 | 35 | 0.35836 | 4.778259277 | complete |
| transcript/33359 | noncoding | 0.0145031 | 35 | 0.41768 | 8.516662598 | complete |
| transcript/33444 | noncoding | 0.00962407 | 35 | 0.29297 | 6.21673584 | complete |
| transcript/33533 | noncoding | 0.0170868 | 35 | 0.41635 | 5.380187988 | complete |
| transcript/33203 | noncoding | 0.0256068 | 35 | 0.48467 | 7.978210449 | incomplete |
| transcript/33324 | noncoding | 0.0152933 | 35 | 0.33716 | 3.916320801 | incomplete |
| transcript/33389 | noncoding | 0.0388316 | 35 | 0.46699 | 8.803283691 | incomplete |
| transcript/33526 | noncoding | 0.017128 | 35 | 0.43853 | 5.203796387 | incomplete |
| transcript/33620 | noncoding | 0.112664 | 35 | 0.45153 | 10.84747314 | incomplete |
| transcript/32999 | noncoding | 0.0112533 | 34 | 0.37882 | 12.84515381 | complete |
| transcript/33148 | noncoding | 0.045932 | 34 | 0.47716 | 9.688171387 | complete |
| transcript/33297 | noncoding | 0.0073785 | 34 | 0.3502 | 6.499694824 | complete |
| transcript/33305 | noncoding | 0.0137824 | 34 | 0.27762 | 5.717102051 | complete |
| transcript/33545 | noncoding | 0.0271016 | 34 | 0.4069 | 4.316101074 | complete |
| transcript/33639 | noncoding | 0.0339787 | 34 | 0.44588 | 10.28448486 | complete |
| transcript/33247 | noncoding | 0.0710634 | 34 | 0.3339 | 7.794616699 | incomplete |
| transcript/33301 | noncoding | 0.0224536 | 34 | 0.36512 | 5.932678223 | incomplete |
| transcript/33455 | noncoding | 0.0163764 | 34 | 0.43107 | 5.704406738 | incomplete |
| transcript/33138 | noncoding | 0.0127434 | 33 | 0.40636 | 5.580383301 | complete |
| transcript/33147 | noncoding | 0.00978881 | 33 | 0.32938 | 9.149475098 | complete |
| transcript/33575 | noncoding | 0.00709021 | 33 | 0.34621 | 7.729187012 | complete |
| transcript/33374 | noncoding | 0.0929727 | 33 | 0.40765 | 10.10565186 | incomplete |
| transcript/33461 | noncoding | 0.0538057 | 33 | 0.41006 | 9.03302002 | incomplete |
| transcript/33483 | noncoding | 0.100472 | 33 | 0.36883 | 9.609558105 | incomplete |
| transcript/33494 | noncoding | 0.0844761 | 33 | 0.36084 | 9.020324707 | incomplete |
| transcript/33605 | noncoding | 0.0380999 | 33 | 0.33982 | 6.48626709 | incomplete |
| transcript/32445 | noncoding | 0.00705859 | 32 | 0.30604 | 7.835144043 | complete |
| transcript/32925 | noncoding | 0.0122819 | 32 | 0.40103 | 9.00592041 | complete |
| transcript/33048 | noncoding | 0.00704674 | 32 | 0.34463 | 7.999084473 | complete |
| transcript/33125 | noncoding | 0.0277315 | 32 | 0.40982 | 4.136901855 | complete |
| transcript/33150 | noncoding | 0.00997107 | 32 | 0.29083 | 9.00592041 | complete |
| transcript/33171 | noncoding | 0.014657 | 32 | 0.45978 | 5.709411621 | complete |
| transcript/33191 | noncoding | 0.0324316 | 32 | 0.43763 | 4.195983887 | complete |
| transcript/33220 | noncoding | 0.00675966 | 32 | 0.36164 | 6.803161621 | complete |
| transcript/33226 | noncoding | 0.0173628 | 32 | 0.37647 | 11.00653076 | complete |
| transcript/33239 | noncoding | 0.0117958 | 32 | 0.37548 | 9.428894043 | complete |
| transcript/33378 | noncoding | 0.0147651 | 32 | 0.41975 | 9.057922363 | complete |
| transcript/33568 | noncoding | 0.0144526 | 32 | 0.37968 | 4.682556152 | complete |
| transcript/33608 | noncoding | 0.0126345 | 32 | 0.41976 | 5.709411621 | complete |
| transcript/33211 | noncoding | 0.0155099 | 32 | 0.41255 | 5.58001709 | incomplete |
| transcript/32791 | noncoding | 0.0120029 | 31 | 0.35291 | 4.535095215 | complete |
| transcript/32903 | noncoding | 0.0212249 | 31 | 0.41478 | 11.83990479 | complete |
| transcript/32924 | noncoding | 0.0189997 | 31 | 0.41987 | 9.992004395 | complete |
| transcript/33031 | noncoding | 0.00799891 | 31 | 0.3475 | 8.675842285 | complete |
| transcript/33100 | noncoding | 0.0344506 | 31 | 0.46724 | 9.788391113 | complete |
| transcript/33105 | noncoding | 0.014192 | 31 | 0.40524 | 9.513122559 | complete |
| transcript/33145 | noncoding | 0.0117214 | 31 | 0.32283 | 4.535095215 | complete |
| transcript/33176 | noncoding | 0.00822915 | 31 | 0.3211 | 8.841491699 | complete |
| transcript/33209 | noncoding | 0.0100235 | 31 | 0.38477 | 8.886169434 | complete |
| transcript/33478 | noncoding | 0.0224544 | 31 | 0.45484 | 4.649353027 | complete |
| transcript/33006 | noncoding | 0.095531 | 31 | 0.30975 | 7.715759277 | incomplete |
| transcript/33285 | noncoding | 0.0472445 | 31 | 0.44262 | 9.150085449 | incomplete |
| transcript/33443 | noncoding | 0.015487 | 31 | 0.41869 | 5.727966309 | incomplete |
| transcript/33550 | noncoding | 0.0164445 | 31 | 0.43071 | 4.515808105 | incomplete |
| transcript/32971 | noncoding | 0.00774521 | 30 | 0.39474 | 7.753356934 | complete |
| transcript/33074 | noncoding | 0.0179828 | 30 | 0.39582 | 4.36907959 | complete |
| transcript/33393 | noncoding | 0.00607374 | 30 | 0.34244 | 7.729553223 | complete |
| transcript/33406 | noncoding | 0.057502 | 30 | 0.47244 | 11.71258545 | complete |
| transcript/33413 | noncoding | 0.0436213 | 30 | 0.46047 | 11.71258545 | complete |
| transcript/33431 | noncoding | 0.0459517 | 30 | 0.46284 | 11.71258545 | complete |
| transcript/33465 | noncoding | 0.0157969 | 30 | 0.41042 | 9.862487793 | complete |
| transcript/33517 | noncoding | 0.00745479 | 30 | 0.32485 | 8.655822754 | complete |
| transcript/33065 | noncoding | 0.0394388 | 30 | 0.39793 | 8.274475098 | incomplete |
| transcript/32790 | noncoding | 0.00743177 | 29 | 0.39162 | 7.956237793 | complete |
| transcript/32939 | noncoding | 0.0291872 | 29 | 0.40078 | 3.665710449 | complete |
| transcript/33072 | noncoding | 0.00620961 | 29 | 0.33239 | 5.823547363 | complete |
| transcript/33136 | noncoding | 0.0168478 | 29 | 0.3859 | 4.207092285 | complete |
| transcript/33261 | noncoding | 0.0165334 | 29 | 0.33738 | 3.665710449 | complete |
| transcript/33289 | noncoding | 0.0127508 | 29 | 0.47011 | 7.65411377 | complete |
| transcript/33362 | noncoding | 0.00635762 | 29 | 0.35976 | 7.999084473 | complete |
| transcript/33402 | noncoding | 0.00786645 | 29 | 0.40098 | 7.914489746 | complete |
| transcript/33404 | noncoding | 0.00793873 | 29 | 0.39994 | 7.999816895 | complete |
| transcript/33582 | noncoding | 0.0085834 | 29 | 0.36408 | 9.034973145 | complete |
| transcript/33600 | noncoding | 0.0071343 | 29 | 0.38518 | 7.955627441 | complete |
| transcript/32956 | noncoding | 0.0166244 | 29 | 0.35711 | 4.952941895 | incomplete |
| transcript/33049 | noncoding | 0.0736147 | 29 | 0.32888 | 7.778747559 | incomplete |
| transcript/33467 | noncoding | 0.0232052 | 29 | 0.31951 | 4.535095215 | incomplete |
| transcript/33635 | noncoding | 0.0148161 | 29 | 0.34809 | 4.351989746 | incomplete |
| transcript/32746 | noncoding | 0.0150294 | 28 | 0.37393 | 11.3036499 | complete |
| transcript/32753 | noncoding | 0.00999928 | 28 | 0.41191 | 8.739074707 | complete |
| transcript/32894 | noncoding | 0.0115533 | 28 | 0.40717 | 9.301940918 | complete |
| transcript/32930 | noncoding | 0.00665248 | 28 | 0.38172 | 7.976745605 | complete |
| transcript/32951 | noncoding | 0.0149922 | 28 | 0.32318 | 11.3036499 | complete |
| transcript/33108 | noncoding | 0.0223566 | 28 | 0.38218 | 3.632629395 | complete |
| transcript/33112 | noncoding | 0.0112327 | 28 | 0.4064 | 9.236999512 | complete |
| transcript/33140 | noncoding | 0.0131842 | 28 | 0.44951 | 8.473449707 | complete |
| transcript/33155 | noncoding | 0.0181404 | 28 | 0.45204 | 4.677062988 | complete |
| transcript/33186 | noncoding | 0.0119726 | 28 | 0.35592 | 10.28289795 | complete |
| transcript/33202 | noncoding | 0.0149342 | 28 | 0.43327 | 9.301452637 | complete |
| transcript/33231 | noncoding | 0.0108666 | 28 | 0.39009 | 9.496276855 | complete |
| transcript/33377 | noncoding | 0.00631665 | 28 | 0.3449 | 5.582946777 | complete |
| transcript/33278 | noncoding | 0.0184826 | 28 | 0.37708 | 5.934875488 | incomplete |
| transcript/33458 | noncoding | 0.0249079 | 28 | 0.45528 | 7.998596191 | incomplete |
| transcript/33471 | noncoding | 0.16456 | 28 | 0.32381 | 9.994689941 | incomplete |
| transcript/33487 | noncoding | 0.0435951 | 28 | 0.41942 | 8.840637207 | incomplete |
| transcript/33500 | noncoding | 0.0172728 | 28 | 0.42847 | 6.692443848 | incomplete |
| transcript/33609 | noncoding | 0.149617 | 28 | 0.30682 | 9.102722168 | incomplete |
| transcript/32781 | noncoding | 0.00521416 | 27 | 0.34131 | 7.697570801 | complete |
| transcript/32825 | noncoding | 0.00524002 | 27 | 0.33911 | 7.737121582 | complete |
| transcript/32912 | noncoding | 0.00575441 | 27 | 0.36296 | 5.949768066 | complete |
| transcript/32913 | noncoding | 0.00559878 | 27 | 0.35574 | 5.937072754 | complete |
| transcript/32916 | noncoding | 0.00932865 | 27 | 0.34459 | 4.493103027 | complete |
| transcript/33063 | noncoding | 0.0119347 | 27 | 0.46611 | 5.270568848 | complete |
| transcript/33081 | noncoding | 0.00642937 | 27 | 0.39338 | 7.697570801 | complete |
| transcript/33153 | noncoding | 0.00604628 | 27 | 0.36978 | 5.89251709 | complete |
| transcript/33163 | noncoding | 0.012104 | 27 | 0.38355 | 4.496887207 | complete |
| transcript/33291 | noncoding | 0.00727141 | 27 | 0.41411 | 6.500793457 | complete |
| transcript/33316 | noncoding | 0.00498813 | 27 | 0.34527 | 6.49432373 | complete |
| transcript/33448 | noncoding | 0.0152382 | 27 | 0.28684 | 4.493103027 | complete |
| transcript/33460 | noncoding | 0.00497032 | 27 | 0.33151 | 6.500793457 | complete |
| transcript/33469 | noncoding | 0.00617864 | 27 | 0.38339 | 7.823791504 | complete |
| transcript/33615 | noncoding | 0.00560088 | 27 | 0.30682 | 7.715393066 | complete |
| transcript/33651 | noncoding | 0.00534609 | 27 | 0.31799 | 7.715393066 | complete |
| transcript/31840 | noncoding | 0.0215804 | 27 | 0.38637 | 6.690734863 | incomplete |
| transcript/32975 | noncoding | 0.168395 | 27 | 0.32017 | 9.994689941 | incomplete |
| transcript/33087 | noncoding | 0.0129729 | 27 | 0.37428 | 4.55645752 | incomplete |
| transcript/33090 | noncoding | 0.0215355 | 27 | 0.48004 | 4.404602051 | incomplete |
| transcript/33299 | noncoding | 0.129301 | 27 | 0.28606 | 7.746154785 | incomplete |
| transcript/33546 | noncoding | 0.0172443 | 27 | 0.33374 | 4.404602051 | incomplete |
| transcript/31827 | noncoding | 0.013211 | 26 | 0.35736 | 11.07513428 | complete |
| transcript/32500 | noncoding | 0.00540591 | 26 | 0.32362 | 5.816345215 | complete |
| transcript/32714 | noncoding | 0.00886364 | 26 | 0.39761 | 12.90789795 | complete |
| transcript/32749 | noncoding | 0.0221817 | 26 | 0.40932 | 3.891296387 | complete |
| transcript/32797 | noncoding | 0.0298045 | 26 | 0.45031 | 3.794616699 | complete |
| transcript/32821 | noncoding | 0.00937137 | 26 | 0.40656 | 12.90789795 | complete |
| transcript/32993 | noncoding | 0.0122659 | 26 | 0.34548 | 3.891296387 | complete |
| transcript/33044 | noncoding | 0.0109788 | 26 | 0.42102 | 9.101379395 | complete |
| transcript/33056 | noncoding | 0.00596398 | 26 | 0.38044 | 7.999450684 | complete |
| transcript/33061 | noncoding | 0.0107118 | 26 | 0.38791 | 9.782043457 | complete |
| transcript/33104 | noncoding | 0.0128651 | 26 | 0.35523 | 3.891296387 | complete |
| transcript/33133 | noncoding | 0.00542341 | 26 | 0.35885 | 7.975891113 | complete |
| transcript/33152 | noncoding | 0.0095665 | 26 | 0.3616 | 9.782043457 | complete |
| transcript/33195 | noncoding | 0.019377 | 26 | 0.39091 | 3.794616699 | complete |
| transcript/33304 | noncoding | 0.0127986 | 26 | 0.35439 | 3.891296387 | complete |
| transcript/33319 | noncoding | 0.00550403 | 26 | 0.31096 | 7.976989746 | complete |
| transcript/33366 | noncoding | 0.00672382 | 26 | 0.29747 | 8.513000488 | complete |
| transcript/33485 | noncoding | 0.0100199 | 26 | 0.35253 | 4.298034668 | complete |
| transcript/33493 | noncoding | 0.0164237 | 26 | 0.42805 | 4.529724121 | complete |
| transcript/33115 | noncoding | 0.0144125 | 26 | 0.42733 | 5.819763184 | incomplete |
| transcript/33281 | noncoding | 0.132945 | 26 | 0.36508 | 10.3180542 | incomplete |
| transcript/32968 | noncoding | 0.0220155 | 25 | 0.4083 | 3.799377441 | complete |
| transcript/32984 | noncoding | 0.0125742 | 25 | 0.40572 | 10.04425049 | complete |
| transcript/33024 | noncoding | 0.00877818 | 25 | 0.30809 | 4.677062988 | complete |
| transcript/33175 | noncoding | 0.0189078 | 25 | 0.42251 | 11.41766357 | complete |
| transcript/33183 | noncoding | 0.0104477 | 25 | 0.4294 | 8.885803223 | complete |
| transcript/33212 | noncoding | 0.0104636 | 25 | 0.43003 | 5.222351074 | complete |
| transcript/33223 | noncoding | 0.00889827 | 25 | 0.39612 | 5.039978027 | complete |
| transcript/33236 | noncoding | 0.0103746 | 25 | 0.25826 | 8.49420166 | complete |
| transcript/33314 | noncoding | 0.00694796 | 25 | 0.4217 | 7.633483887 | complete |
| transcript/33330 | noncoding | 0.00711894 | 25 | 0.42925 | 6.481262207 | complete |
| transcript/33345 | noncoding | 0.0143724 | 25 | 0.39555 | 4.207092285 | complete |
| transcript/33420 | noncoding | 0.0173514 | 25 | 0.43644 | 10.04425049 | complete |
| transcript/33492 | noncoding | 0.0121465 | 25 | 0.34358 | 3.794616699 | complete |
| transcript/33499 | noncoding | 0.0100807 | 25 | 0.42362 | 5.222351074 | complete |
| transcript/33564 | noncoding | 0.0132185 | 25 | 0.36505 | 11.41766357 | complete |
| transcript/33185 | noncoding | 0.0273335 | 25 | 0.33687 | 5.745422363 | incomplete |
| transcript/33253 | noncoding | 0.0145798 | 25 | 0.44983 | 5.935119629 | incomplete |
| transcript/33315 | noncoding | 0.016791 | 25 | 0.44879 | 4.529602051 | incomplete |
| transcript/33456 | noncoding | 0.0339551 | 25 | 0.44047 | 8.635192871 | incomplete |
| transcript/33470 | noncoding | 0.10959 | 25 | 0.32167 | 8.611755371 | incomplete |
| transcript/33604 | noncoding | 0.0174513 | 25 | 0.40249 | 6.497497559 | incomplete |
| transcript/33078 | noncoding | 0.0238272 | 24 | 0.44253 | 11.55377197 | complete |
| transcript/33092 | noncoding | 0.008583 | 24 | 0.29134 | 9.349304199 | complete |
| transcript/33225 | noncoding | 0.0139396 | 24 | 0.41096 | 10.41033936 | complete |
| transcript/33255 | noncoding | 0.00599727 | 24 | 0.41594 | 6.692687988 | complete |
| transcript/33351 | noncoding | 0.00658407 | 24 | 0.41365 | 7.976989746 | complete |
| transcript/33473 | noncoding | 0.01148 | 24 | 0.32699 | 3.799377441 | complete |
| transcript/33504 | noncoding | 0.00453262 | 24 | 0.37107 | 6.69354248 | complete |
| transcript/33563 | noncoding | 0.0124726 | 24 | 0.39846 | 4.36907959 | complete |
| transcript/33606 | noncoding | 0.0117521 | 24 | 0.37784 | 4.136901855 | complete |
| transcript/32792 | noncoding | 0.0257673 | 24 | 0.31525 | 4.745788574 | incomplete |
| transcript/33098 | noncoding | 0.0283515 | 24 | 0.41735 | 8.039489746 | incomplete |
| transcript/33146 | noncoding | 0.00953888 | 24 | 0.3721 | 3.373962402 | incomplete |
| transcript/33553 | noncoding | 0.0186203 | 24 | 0.34415 | 5.059997559 | incomplete |
| transcript/32355 | noncoding | 0.00730568 | 23 | 0.43472 | 7.956848145 | complete |
| transcript/32879 | noncoding | 0.00644655 | 23 | 0.41909 | 7.956848145 | complete |
| transcript/33015 | noncoding | 0.0188449 | 23 | 0.39739 | 3.665710449 | complete |
| transcript/33021 | noncoding | 0.00957719 | 23 | 0.36048 | 4.129821777 | complete |
| transcript/33162 | noncoding | 0.00532127 | 23 | 0.40484 | 6.500793457 | complete |
| transcript/33180 | noncoding | 0.00654561 | 23 | 0.42769 | 7.71295166 | complete |
| transcript/33181 | noncoding | 0.00749667 | 23 | 0.42212 | 5.585144043 | complete |
| transcript/33217 | noncoding | 0.00453931 | 23 | 0.34578 | 7.956848145 | complete |
| transcript/33320 | noncoding | 0.00531811 | 23 | 0.3819 | 5.753234863 | complete |
| transcript/33370 | noncoding | 0.0100306 | 23 | 0.34636 | 3.925598145 | complete |
| transcript/33382 | noncoding | 0.00438695 | 23 | 0.32819 | 5.969299316 | complete |
| transcript/33416 | noncoding | 0.00753799 | 23 | 0.43349 | 5.750793457 | complete |
| transcript/33454 | noncoding | 0.00474019 | 23 | 0.31523 | 7.956848145 | complete |
| transcript/33540 | noncoding | 0.00971192 | 23 | 0.48643 | 4.538635254 | complete |
| transcript/33607 | noncoding | 0.00477845 | 23 | 0.36827 | 7.953796387 | complete |
| transcript/33385 | noncoding | 0.0207631 | 23 | 0.45698 | 7.737976074 | incomplete |
| transcript/33507 | noncoding | 0.0117785 | 23 | 0.40086 | 3.492248535 | incomplete |
| transcript/33633 | noncoding | 0.101918 | 23 | 0.30499 | 7.810241699 | incomplete |
| transcript/32635 | noncoding | 0.0117881 | 22 | 0.40909 | 10.21258545 | complete |
| transcript/32729 | noncoding | 0.00507207 | 22 | 0.37634 | 8.274353027 | complete |
| transcript/32834 | noncoding | 0.00909964 | 22 | 0.43259 | 5.125549316 | complete |
| transcript/32852 | noncoding | 0.0118824 | 22 | 0.41004 | 10.21258545 | complete |
| transcript/32864 | noncoding | 0.00745391 | 22 | 0.37112 | 9.489196777 | complete |
| transcript/32976 | noncoding | 0.00615095 | 22 | 0.30443 | 8.842346191 | complete |
| transcript/32986 | noncoding | 0.0100404 | 22 | 0.38419 | 10.21258545 | complete |
| transcript/33011 | noncoding | 0.0110577 | 22 | 0.39008 | 10.4463501 | complete |
| transcript/33022 | noncoding | 0.00713129 | 22 | 0.3512 | 9.489196777 | complete |
| transcript/33037 | noncoding | 0.0109696 | 22 | 0.41727 | 9.778991699 | complete |
| transcript/33106 | noncoding | 0.00948709 | 22 | 0.45099 | 5.125549316 | complete |
| transcript/33119 | noncoding | 0.0316695 | 22 | 0.46884 | 12.30426025 | complete |
| transcript/33123 | noncoding | 0.00634899 | 22 | 0.4465 | 6.699279785 | complete |
| transcript/33141 | noncoding | 0.00729216 | 22 | 0.44967 | 5.821105957 | complete |
| transcript/33157 | noncoding | 0.00634307 | 22 | 0.40484 | 8.498474121 | complete |
| transcript/33190 | noncoding | 0.00757399 | 22 | 0.37557 | 9.489196777 | complete |
| transcript/33200 | noncoding | 0.00693374 | 22 | 0.43499 | 7.976989746 | complete |
| transcript/33204 | noncoding | 0.00606926 | 22 | 0.40785 | 8.274353027 | complete |
| transcript/33243 | noncoding | 0.00914359 | 22 | 0.39914 | 4.674255371 | complete |
| transcript/33294 | noncoding | 0.00719975 | 22 | 0.35866 | 4.515686035 | complete |
| transcript/33327 | noncoding | 0.00617331 | 22 | 0.42747 | 7.728210449 | complete |
| transcript/33369 | noncoding | 0.00421631 | 22 | 0.3385 | 5.818786621 | complete |
| transcript/33417 | noncoding | 0.00591988 | 22 | 0.40508 | 8.253723145 | complete |
| transcript/33419 | noncoding | 0.00563758 | 22 | 0.42259 | 6.490905762 | complete |
| transcript/33423 | noncoding | 0.00397146 | 22 | 0.32111 | 6.487976074 | complete |
| transcript/33439 | noncoding | 0.00814644 | 22 | 0.32052 | 9.778991699 | complete |
| transcript/33441 | noncoding | 0.00670923 | 22 | 0.46644 | 6.480529785 | complete |
| transcript/33552 | noncoding | 0.00816337 | 22 | 0.45549 | 7.953918457 | complete |
| transcript/33580 | noncoding | 0.00772923 | 22 | 0.4171 | 5.274963379 | complete |
| transcript/33593 | noncoding | 0.0131219 | 22 | 0.39787 | 4.084533691 | complete |
| transcript/33595 | noncoding | 0.0156569 | 22 | 0.41585 | 4.084533691 | complete |
| transcript/33610 | noncoding | 0.00635297 | 22 | 0.41146 | 8.343688965 | complete |
| transcript/33099 | noncoding | 0.0128163 | 22 | 0.4206 | 5.060119629 | incomplete |
| transcript/33241 | noncoding | 0.0137296 | 22 | 0.43009 | 5.975402832 | incomplete |
| transcript/33591 | noncoding | 0.0188507 | 22 | 0.33529 | 4.846252441 | incomplete |
| transcript/33597 | noncoding | 0.0202645 | 22 | 0.36199 | 5.975402832 | incomplete |
| transcript/33652 | noncoding | 0.015503 | 22 | 0.43574 | 6.692199707 | incomplete |
| transcript/32796 | noncoding | 0.00638255 | 21 | 0.31873 | 4.677062988 | complete |
| transcript/33043 | noncoding | 0.00435708 | 21 | 0.31714 | 7.999816895 | complete |
| transcript/33116 | noncoding | 0.0138244 | 21 | 0.44857 | 4.36907959 | complete |
| transcript/33117 | noncoding | 0.00713111 | 21 | 0.37875 | 4.682556152 | complete |
| transcript/33121 | noncoding | 0.00468796 | 21 | 0.38591 | 7.998840332 | complete |
| transcript/33205 | noncoding | 0.0195585 | 21 | 0.45027 | 3.896057129 | complete |
| transcript/33250 | noncoding | 0.00718721 | 21 | 0.42066 | 8.681945801 | complete |
| transcript/33267 | noncoding | 0.0135375 | 21 | 0.36918 | 3.492248535 | complete |
| transcript/33398 | noncoding | 0.0050688 | 21 | 0.38338 | 8.345397949 | complete |
| transcript/33438 | noncoding | 0.00387132 | 21 | 0.33833 | 5.961975098 | complete |
| transcript/33490 | noncoding | 0.00775263 | 21 | 0.38078 | 9.624694824 | complete |
| transcript/33542 | noncoding | 0.00593508 | 21 | 0.34047 | 4.677062988 | complete |
| transcript/33585 | noncoding | 0.00536204 | 21 | 0.39322 | 5.584899902 | complete |
| transcript/32839 | noncoding | 0.0144889 | 21 | 0.46522 | 5.302185059 | incomplete |
| transcript/33262 | noncoding | 0.0359404 | 21 | 0.33617 | 6.487609863 | incomplete |
| transcript/33333 | noncoding | 0.0174719 | 21 | 0.37462 | 5.984313965 | incomplete |
| transcript/32955 | noncoding | 0.00717801 | 20 | 0.38421 | 9.500671387 | complete |
| transcript/32983 | noncoding | 0.00511068 | 20 | 0.35863 | 8.78692627 | complete |
| transcript/33013 | noncoding | 0.00398966 | 20 | 0.36996 | 5.895446777 | complete |
| transcript/33042 | noncoding | 0.00724379 | 20 | 0.42315 | 8.802307129 | complete |
| transcript/33083 | noncoding | 0.00511431 | 20 | 0.41199 | 5.969299316 | complete |
| transcript/33110 | noncoding | 0.0129473 | 20 | 0.39054 | 3.799377441 | complete |
| transcript/33309 | noncoding | 0.0107486 | 20 | 0.35413 | 11.12213135 | complete |
| transcript/33364 | noncoding | 0.00427752 | 20 | 0.30287 | 6.198547363 | complete |
| transcript/33405 | noncoding | 0.00474117 | 20 | 0.3011 | 5.822814941 | complete |
| transcript/33429 | noncoding | 0.00446417 | 20 | 0.3985 | 7.692443848 | complete |
| transcript/33442 | noncoding | 0.00766779 | 20 | 0.391 | 4.625061035 | complete |
| transcript/33462 | noncoding | 0.0166755 | 20 | 0.28606 | 3.799377441 | complete |
| transcript/33495 | noncoding | 0.00740196 | 20 | 0.46695 | 5.270324707 | complete |
| transcript/33519 | noncoding | 0.0101636 | 20 | 0.33863 | 3.563415527 | complete |
| transcript/33536 | noncoding | 0.00422208 | 20 | 0.38411 | 7.809997559 | complete |
| transcript/33574 | noncoding | 0.00700134 | 20 | 0.43069 | 8.516662598 | complete |
| transcript/32393 | noncoding | 0.0629781 | 20 | 0.39462 | 9.303649902 | incomplete |
| transcript/33007 | noncoding | 0.0462352 | 20 | 0.3699 | 8.159973145 | incomplete |
| transcript/33142 | noncoding | 0.0543071 | 20 | 0.45434 | 9.69720459 | incomplete |
| transcript/33246 | noncoding | 0.175721 | 20 | 0.22907 | 5.218933105 | incomplete |
| transcript/33414 | noncoding | 0.0107616 | 20 | 0.38814 | 4.207092285 | incomplete |
| transcript/32982 | noncoding | 0.00463533 | 19 | 0.39799 | 5.750549316 | complete |
| transcript/33057 | noncoding | 0.00548873 | 19 | 0.46051 | 6.488342285 | complete |
| transcript/33179 | noncoding | 0.0042532 | 19 | 0.39026 | 5.869445801 | complete |
| transcript/33210 | noncoding | 0.00886417 | 19 | 0.40422 | 9.901062012 | complete |
| transcript/33237 | noncoding | 0.00439438 | 19 | 0.41331 | 6.49822998 | complete |
| transcript/33240 | noncoding | 0.00572266 | 19 | 0.30093 | 8.886657715 | complete |
| transcript/33292 | noncoding | 0.0086306 | 19 | 0.34039 | 10.347229 | complete |
| transcript/33308 | noncoding | 0.00751752 | 19 | 0.30736 | 9.69708252 | complete |
| transcript/33343 | noncoding | 0.00667714 | 19 | 0.41632 | 8.886657715 | complete |
| transcript/33348 | noncoding | 0.00788497 | 19 | 0.43017 | 9.00592041 | complete |
| transcript/33375 | noncoding | 0.00864054 | 19 | 0.4506 | 8.71295166 | complete |
| transcript/33475 | noncoding | 0.00634504 | 19 | 0.44243 | 5.580627441 | complete |
| transcript/33498 | noncoding | 0.0054767 | 19 | 0.38031 | 8.938903809 | complete |
| transcript/33508 | noncoding | 0.00502361 | 19 | 0.35463 | 8.886657715 | complete |
| transcript/33512 | noncoding | 0.00406339 | 19 | 0.29792 | 6.685852051 | complete |
| transcript/33537 | noncoding | 0.00363162 | 19 | 0.34952 | 7.821716309 | complete |
| transcript/33601 | noncoding | 0.00359675 | 19 | 0.38007 | 6.484069824 | complete |
| transcript/33602 | noncoding | 0.00500727 | 19 | 0.3514 | 8.886657715 | complete |
| transcript/33234 | noncoding | 0.0134939 | 19 | 0.40555 | 5.897888184 | incomplete |
| transcript/33363 | noncoding | 0.0460377 | 19 | 0.42262 | 9.151062012 | incomplete |
| transcript/33367 | noncoding | 0.0642994 | 19 | 0.38564 | 9.187805176 | incomplete |
| transcript/33376 | noncoding | 0.0125584 | 19 | 0.41306 | 5.582946777 | incomplete |
| transcript/33653 | noncoding | 0.0993321 | 19 | 0.35907 | 9.500061035 | incomplete |
| transcript/32895 | noncoding | 0.0042755 | 18 | 0.40328 | 5.946228027 | complete |
| transcript/33026 | noncoding | 0.00458059 | 18 | 0.41366 | 5.946228027 | complete |
| transcript/33079 | noncoding | 0.0037547 | 18 | 0.39835 | 6.50567627 | complete |
| transcript/33103 | noncoding | 0.00609141 | 18 | 0.42036 | 5.268371582 | complete |
| transcript/33279 | noncoding | 0.00340601 | 18 | 0.36297 | 5.946228027 | complete |
| transcript/33310 | noncoding | 0.00627956 | 18 | 0.43206 | 8.498474121 | complete |
| transcript/33313 | noncoding | 0.00424279 | 18 | 0.37672 | 8.36505127 | complete |
| transcript/33373 | noncoding | 0.00896128 | 18 | 0.39897 | 12.47906494 | complete |
| transcript/33396 | noncoding | 0.00546926 | 18 | 0.33076 | 4.50567627 | complete |
| transcript/33432 | noncoding | 0.00401851 | 18 | 0.32937 | 5.270080566 | complete |
| transcript/33513 | noncoding | 0.00626492 | 18 | 0.37456 | 4.50567627 | complete |
| transcript/33549 | noncoding | 0.00319218 | 18 | 0.36619 | 6.50189209 | complete |
| transcript/33557 | noncoding | 0.00366463 | 18 | 0.33008 | 7.999938965 | complete |
| transcript/33596 | noncoding | 0.00471829 | 18 | 0.31402 | 8.655578613 | complete |
| transcript/33623 | noncoding | 0.00366401 | 18 | 0.3613 | 7.99798584 | complete |
| transcript/33445 | noncoding | 0.0145917 | 18 | 0.36332 | 5.222595215 | incomplete |
| transcript/33477 | noncoding | 0.104693 | 18 | 0.39336 | 10.347229 | incomplete |
| transcript/33527 | noncoding | 0.0355725 | 18 | 0.37708 | 7.846862793 | incomplete |
| transcript/32942 | noncoding | 0.00371413 | 17 | 0.34418 | 8.275939941 | complete |
| transcript/32950 | noncoding | 0.00412025 | 17 | 0.42336 | 6.516540527 | complete |
| transcript/33051 | noncoding | 0.00947032 | 17 | 0.44613 | 9.303527832 | complete |
| transcript/33296 | noncoding | 0.00830698 | 17 | 0.38731 | 12.47906494 | complete |
| transcript/33312 | noncoding | 0.00551744 | 17 | 0.37061 | 9.303527832 | complete |
| transcript/33390 | noncoding | 0.00341503 | 17 | 0.39287 | 6.520202637 | complete |
| transcript/33407 | noncoding | 0.00371891 | 17 | 0.34861 | 5.272766113 | complete |
| transcript/33410 | noncoding | 0.00555396 | 17 | 0.37306 | 9.303527832 | complete |
| transcript/33436 | noncoding | 0.00311793 | 17 | 0.37483 | 6.49786377 | complete |
| transcript/33463 | noncoding | 0.012351 | 17 | 0.43112 | 10.4463501 | complete |
| transcript/33464 | noncoding | 0.0110688 | 17 | 0.39431 | 3.799377441 | complete |
| transcript/33479 | noncoding | 0.0119797 | 17 | 0.28783 | 3.998840332 | complete |
| transcript/33524 | noncoding | 0.00393681 | 17 | 0.37763 | 5.451965332 | complete |
| transcript/33560 | noncoding | 0.00636672 | 17 | 0.31786 | 4.237121582 | complete |
| transcript/33578 | noncoding | 0.00410526 | 17 | 0.38913 | 8.254577637 | complete |
| transcript/33587 | noncoding | 0.00337579 | 17 | 0.34143 | 5.580871582 | complete |
| transcript/33592 | noncoding | 0.00587824 | 17 | 0.34935 | 4.239562988 | complete |
| transcript/33619 | noncoding | 0.00598667 | 17 | 0.39207 | 9.303527832 | complete |
| transcript/33621 | noncoding | 0.00798546 | 17 | 0.43052 | 9.303527832 | complete |
| transcript/33624 | noncoding | 0.00318422 | 17 | 0.34406 | 5.820617676 | complete |
| transcript/33630 | noncoding | 0.0095691 | 17 | 0.447 | 9.303527832 | complete |
| transcript/33503 | noncoding | 0.0711026 | 17 | 0.32608 | 7.761657715 | incomplete |
| transcript/33509 | noncoding | 0.0233744 | 17 | 0.43015 | 7.975524902 | incomplete |
| transcript/33611 | noncoding | 0.0732078 | 17 | 0.39841 | 9.700378418 | incomplete |
| transcript/33628 | noncoding | 0.147976 | 17 | 0.28195 | 8.189758301 | incomplete |
| transcript/33643 | noncoding | 0.0619466 | 17 | 0.39399 | 9.303527832 | incomplete |
| transcript/33091 | noncoding | 0.00403041 | 16 | 0.37781 | 5.222106934 | complete |
| transcript/33168 | noncoding | 0.0034929 | 16 | 0.34356 | 5.274597168 | complete |
| transcript/33232 | noncoding | 0.00946685 | 16 | 0.3107 | 3.563415527 | complete |
| transcript/33488 | noncoding | 0.00756994 | 16 | 0.41296 | 9.699645996 | complete |
| transcript/33496 | noncoding | 0.0100049 | 16 | 0.40142 | 12.01654053 | complete |
| transcript/33520 | noncoding | 0.00388655 | 16 | 0.3303 | 8.498474121 | complete |
| transcript/33531 | noncoding | 0.00647139 | 16 | 0.28767 | 9.188293457 | complete |
| transcript/33570 | noncoding | 0.00327784 | 16 | 0.37858 | 5.897644043 | complete |
| transcript/33618 | noncoding | 0.009804 | 16 | 0.32769 | 11.00018311 | complete |
| transcript/32890 | noncoding | 0.0152618 | 16 | 0.40332 | 6.491394043 | incomplete |
| transcript/33365 | noncoding | 0.0135829 | 16 | 0.40127 | 6.039733887 | incomplete |
| transcript/33425 | noncoding | 0.0265147 | 16 | 0.41104 | 7.999816895 | incomplete |
| transcript/33586 | noncoding | 0.0489066 | 16 | 0.42182 | 9.303527832 | incomplete |
| transcript/33626 | noncoding | 0.107757 | 16 | 0.31298 | 8.344665527 | incomplete |
| transcript/32964 | noncoding | 0.0134464 | 15 | 0.46622 | 9.700256348 | complete |
| transcript/32974 | noncoding | 0.00709715 | 15 | 0.44473 | 8.93951416 | complete |
| transcript/33077 | noncoding | 0.0138903 | 15 | 0.44993 | 10.34698486 | complete |
| transcript/33134 | noncoding | 0.00407771 | 15 | 0.39418 | 8.498474121 | complete |
| transcript/33165 | noncoding | 0.00264015 | 15 | 0.35892 | 6.499816895 | complete |
| transcript/33228 | noncoding | 0.00360927 | 15 | 0.32389 | 8.344665527 | complete |
| transcript/33229 | noncoding | 0.00502818 | 15 | 0.32538 | 4.36907959 | complete |
| transcript/33318 | noncoding | 0.00365361 | 15 | 0.35937 | 8.498474121 | complete |
| transcript/33326 | noncoding | 0.00452142 | 15 | 0.45469 | 5.895202637 | complete |
| transcript/33459 | noncoding | 0.00363159 | 15 | 0.34944 | 8.498474121 | complete |
| transcript/33466 | noncoding | 0.00376205 | 15 | 0.37374 | 8.498474121 | complete |
| transcript/33535 | noncoding | 0.0128978 | 15 | 0.40499 | 3.574279785 | complete |
| transcript/33541 | noncoding | 0.00368185 | 15 | 0.36448 | 8.498474121 | complete |
| transcript/33543 | noncoding | 0.0033975 | 15 | 0.35968 | 5.270324707 | complete |
| transcript/33637 | noncoding | 0.0102128 | 15 | 0.45402 | 9.496643066 | complete |
| transcript/32712 | noncoding | 0.00708423 | 14 | 0.36988 | 3.799377441 | complete |
| transcript/32893 | noncoding | 0.0100736 | 14 | 0.43008 | 4.084533691 | complete |
| transcript/32940 | noncoding | 0.00749878 | 14 | 0.37614 | 3.799377441 | complete |
| transcript/32944 | noncoding | 0.0106708 | 14 | 0.40983 | 3.799377441 | complete |
| transcript/33016 | noncoding | 0.00406502 | 14 | 0.43568 | 5.874206543 | complete |
| transcript/33084 | noncoding | 0.0125662 | 14 | 0.45464 | 10.05218506 | complete |
| transcript/33193 | noncoding | 0.00289769 | 14 | 0.36686 | 7.83404541 | complete |
| transcript/33274 | noncoding | 0.00282566 | 14 | 0.34421 | 7.799133301 | complete |
| transcript/33381 | noncoding | 0.00811672 | 14 | 0.38403 | 3.799377441 | complete |
| transcript/33457 | noncoding | 0.00487258 | 14 | 0.34806 | 4.239562988 | complete |
| transcript/33571 | noncoding | 0.00284049 | 14 | 0.36029 | 7.799133301 | complete |
| transcript/33573 | noncoding | 0.0098516 | 14 | 0.43655 | 10.05218506 | complete |
| transcript/33583 | noncoding | 0.00329192 | 14 | 0.367 | 5.272521973 | complete |
| transcript/33584 | noncoding | 0.00307394 | 14 | 0.38262 | 5.753234863 | complete |
| transcript/33613 | noncoding | 0.00852159 | 14 | 0.35302 | 11.00030518 | complete |
| transcript/33614 | noncoding | 0.00383822 | 14 | 0.42562 | 7.799133301 | complete |
| transcript/33627 | noncoding | 0.00383211 | 14 | 0.40087 | 8.364807129 | complete |
| transcript/33082 | noncoding | 0.0697348 | 14 | 0.42835 | 10.05218506 | incomplete |
| transcript/33358 | noncoding | 0.0181207 | 14 | 0.46121 | 3.925598145 | incomplete |
| transcript/33474 | noncoding | 0.0136316 | 14 | 0.38355 | 5.753234863 | incomplete |
| transcript/33486 | noncoding | 0.0354737 | 14 | 0.39608 | 8.344665527 | incomplete |
| transcript/33622 | noncoding | 0.0246316 | 14 | 0.41763 | 7.999816895 | incomplete |
| transcript/33172 | noncoding | 0.00872012 | 13 | 0.39552 | 11.00030518 | complete |
| transcript/33265 | noncoding | 0.00315798 | 13 | 0.37122 | 5.274719238 | complete |
| transcript/33482 | noncoding | 0.00577252 | 13 | 0.34157 | 3.799377441 | complete |
| transcript/33502 | noncoding | 0.00285776 | 13 | 0.33851 | 7.999816895 | complete |
| transcript/33551 | noncoding | 0.00461271 | 13 | 0.37418 | 9.302307129 | complete |
| transcript/33558 | noncoding | 0.00278184 | 13 | 0.39911 | 6.495300293 | complete |
| transcript/33649 | noncoding | 0.00280951 | 13 | 0.36762 | 5.587219238 | complete |
| transcript/33017 | noncoding | 0.0182301 | 13 | 0.35479 | 5.753234863 | incomplete |
| transcript/33131 | noncoding | 0.0115009 | 13 | 0.41513 | 3.492248535 | incomplete |
| transcript/33562 | noncoding | 0.0119861 | 13 | 0.47264 | 5.934875488 | incomplete |
| transcript/33631 | noncoding | 0.0973133 | 13 | 0.36693 | 9.700256348 | incomplete |
| transcript/33636 | noncoding | 0.0129624 | 13 | 0.3884 | 5.748474121 | incomplete |
| transcript/32952 | noncoding | 0.0100855 | 12 | 0.43442 | 3.929626465 | complete |
| transcript/33379 | noncoding | 0.00253232 | 12 | 0.34954 | 5.587219238 | complete |
| transcript/33394 | noncoding | 0.0074486 | 12 | 0.44137 | 4.36907959 | complete |
| transcript/33476 | noncoding | 0.0071359 | 12 | 0.38435 | 3.799377441 | complete |
| transcript/33539 | noncoding | 0.00253679 | 12 | 0.34174 | 5.58770752 | complete |
| transcript/33554 | noncoding | 0.00336089 | 12 | 0.40866 | 5.586975098 | complete |
| transcript/33656 | noncoding | 0.00326318 | 12 | 0.40406 | 5.585388184 | complete |
| transcript/33556 | noncoding | 0.0490239 | 12 | 0.43308 | 9.500061035 | incomplete |
| transcript/33581 | noncoding | 0.0108829 | 12 | 0.42179 | 5.272766113 | incomplete |
| transcript/33645 | noncoding | 0.0485957 | 12 | 0.37444 | 8.500061035 | incomplete |
| transcript/33256 | noncoding | 1.79E-05 | 11 | 0.37223 | 5.752746582 | complete |
| transcript/33349 | noncoding | 0.00687052 | 11 | 0.40663 | 4.084533691 | complete |
| transcript/33408 | noncoding | 0.0038675 | 11 | 0.42216 | 5.274353027 | complete |
| transcript/33424 | noncoding | 0.00919376 | 11 | 0.46832 | 9.349304199 | complete |
| transcript/33433 | noncoding | 0.0072131 | 11 | 0.26299 | 5.753234863 | complete |
| transcript/33451 | noncoding | 0.00292111 | 11 | 0.37424 | 8.345397949 | complete |
| transcript/33491 | noncoding | 0.00281043 | 11 | 0.37248 | 5.274963379 | complete |
| transcript/33577 | noncoding | 0.00322501 | 11 | 0.45509 | 6.683898926 | complete |
| transcript/33632 | noncoding | 0.003304 | 11 | 0.39811 | 5.272766113 | complete |
| transcript/33372 | noncoding | 0.0847686 | 11 | 0.3191 | 7.999816895 | incomplete |
| transcript/33412 | noncoding | 0.0238282 | 11 | 0.36528 | 6.806091309 | incomplete |
| transcript/33647 | noncoding | 0.0332629 | 11 | 0.30674 | 5.272766113 | incomplete |
| transcript/33384 | noncoding | 0.00372271 | 10 | 0.44775 | 7.975524902 | complete |
| transcript/33399 | noncoding | 0.00430592 | 10 | 0.33998 | 3.998840332 | complete |
| transcript/33510 | noncoding | 0.00485478 | 10 | 0.48358 | 7.999816895 | complete |
| transcript/33521 | noncoding | 0.00392297 | 10 | 0.45617 | 5.272766113 | complete |
| transcript/33538 | noncoding | 0.00322211 | 10 | 0.42692 | 7.999816895 | complete |
| transcript/33589 | noncoding | 0.0033769 | 10 | 0.45825 | 5.713928223 | complete |
| transcript/33655 | noncoding | 0.00480473 | 10 | 0.3389 | 3.799377441 | complete |
| transcript/33544 | noncoding | 0.119668 | 10 | 0.40798 | 11.00030518 | incomplete |
| transcript/33640 | noncoding | 0.0173398 | 10 | 0.36211 | 5.969299316 | incomplete |
| transcript/33650 | noncoding | 0.00946895 | 10 | 0.35115 | 3.998840332 | incomplete |
| transcript/33154 | noncoding | 0.00270255 | 9 | 0.40475 | 5.58770752 | complete |
| transcript/33182 | noncoding | 0.00320162 | 9 | 0.40964 | 8.500061035 | complete |
| transcript/33219 | noncoding | 0.00412932 | 9 | 0.44389 | 8.500061035 | complete |
| transcript/33335 | noncoding | 0.00264106 | 9 | 0.33945 | 8.344665527 | complete |
| transcript/33523 | noncoding | 0.00518587 | 9 | 0.3713 | 3.799377441 | complete |
| transcript/33534 | noncoding | 0.0026957 | 9 | 0.33234 | 8.344665527 | complete |
| transcript/33559 | noncoding | 0.0030944 | 9 | 0.30743 | 8.344665527 | complete |
| transcript/33566 | noncoding | 0.00713152 | 9 | 0.39676 | 10.83441162 | complete |
| transcript/33590 | noncoding | 0.00821804 | 9 | 0.41431 | 11.00030518 | complete |
| transcript/33616 | noncoding | 0.00364823 | 9 | 0.45777 | 5.274963379 | complete |
| transcript/33629 | noncoding | 1.83E-05 | 9 | 0.37075 | 5.274963379 | complete |
| transcript/33634 | noncoding | 0.00258864 | 9 | 0.35841 | 8.344665527 | complete |
| transcript/33642 | noncoding | 0.00986943 | 9 | 0.33614 | 3.799377441 | incomplete |
| transcript/33646 | noncoding | 0.0358242 | 9 | 0.36923 | 7.821716309 | incomplete |
| transcript/33264 | noncoding | 1.68E-05 | 8 | 0.37454 | 5.272766113 | complete |
| transcript/33357 | noncoding | 1.45E-05 | 8 | 0.34882 | 5.274719238 | complete |
| transcript/33561 | noncoding | 0.00258811 | 8 | 0.39052 | 5.274963379 | complete |
| transcript/33648 | noncoding | 1.51E-05 | 8 | 0.3606 | 5.270324707 | complete |
| transcript/33392 | noncoding | 0.0142117 | 8 | 0.35471 | 5.274719238 | incomplete |
| transcript/33612 | noncoding | 0.00796322 | 8 | 0.37509 | 3.799377441 | incomplete |
| transcript/32967 | noncoding | 0.0027078 | 7 | 0.40715 | 5.274963379 | complete |
| transcript/32972 | noncoding | 1.29E-05 | 7 | 0.34803 | 5.274963379 | complete |
| transcript/33030 | noncoding | 1.63E-05 | 7 | 0.37999 | 5.222351074 | complete |
| transcript/33214 | noncoding | 0.00349839 | 7 | 0.39212 | 4.529724121 | complete |
| transcript/33355 | noncoding | 1.56E-05 | 7 | 0.32227 | 5.274719238 | complete |
| transcript/33428 | noncoding | 0.00274318 | 7 | 0.45417 | 5.752990723 | complete |
| transcript/33472 | noncoding | 0.00322024 | 7 | 0.44674 | 5.274719238 | complete |
| transcript/33644 | noncoding | 0.00409577 | 7 | 0.36467 | 9.700256348 | complete |
| transcript/33657 | noncoding | 0.00421787 | 7 | 0.34262 | 9.700256348 | complete |
| transcript/32641 | noncoding | 0.010154 | 7 | 0.43045 | 5.274719238 | incomplete |
| transcript/33293 | noncoding | 0.0106342 | 7 | 0.42141 | 3.799377441 | incomplete |
| transcript/33268 | noncoding | 1.21E-05 | 6 | 0.33485 | 5.274963379 | complete |
| transcript/33391 | noncoding | 0.00362867 | 6 | 0.35224 | 9.500061035 | complete |
| transcript/33530 | noncoding | 1.24E-05 | 6 | 0.33198 | 5.274963379 | complete |
| transcript/33594 | noncoding | 0.00292748 | 6 | 0.46234 | 5.274719238 | complete |
| transcript/33617 | noncoding | 1.20E-05 | 5 | 0.37868 | 5.274963379 | complete |
| transcript/32777 | noncoding | 0.0033468 | 4 | 0.41735 | 4.596496582 | complete |
| transcript/33169 | noncoding | 0.00372633 | 4 | 0.44486 | 4.596496582 | complete |
| transcript/33555 | noncoding | 1.41E-05 | 4 | 0.40242 | 5.274719238 | complete |
| transcript/33453 | noncoding | 0.00974555 | 4 | 0.43516 | 5.272521973 | incomplete |
| transcript/33422 | noncoding | 0.00947017 | 3 | 0.43059 | 5.274963379 | incomplete |
| transcript/33403 | noncoding | 1.59E-05 | 2 | 0.45323 | 5.274963379 | complete |
| transcript/33641 | noncoding | 9.08E-06 | 2 | 0.3875 | 5.274963379 | complete |
| transcript/33522 | noncoding | 0.042392 | 2 | 0.29152 | 5.274963379 | incomplete |
| transcript/32843 | noncoding | 0.0149873 | 0 | 0.42447 | 0 | incomplete |
| transcript/33340 | noncoding | 5.14E-07 | 0 | 0.32388 | 0 | incomplete |
| transcript/33360 | noncoding | 1.32E-05 | 0 | 0.38348 | 0 | incomplete |
| transcript/33567 | noncoding | 2.24E-06 | 0 | 0.35994 | 0 | incomplete |
| transcript/33603 | noncoding | 0.00352222 | 0 | 0.39504 | 0 | incomplete |
| transcript/33625 | noncoding | 5.28E-07 | 0 | 0.32542 | 0 | incomplete |
