# Supplementary figures and images for: The Long Read Transcriptome of Rice (Oryza sativa ssp. japonica var. Nipponbare) Reveals Novel Transcripts
Source: Rice (N Y). 2022 Jun 11;15:29. doi: 10.1186/s12284-022-00577-1 (PMC9188635; doi:10.1186/s12284-022-00577-1)

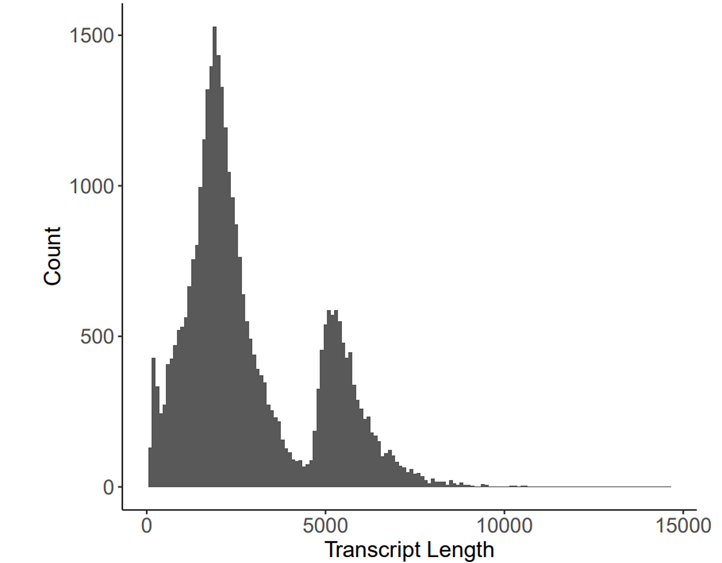

Supplement: Supplementary file 3 — Additional file 3. Fig S1: Length distribution of all FLNC transcripts. Two peaks corresponding to two library bin sizes of > 4kb and < 4kb. [file 12284_2022_577_MOESM3_ESM.jpg]

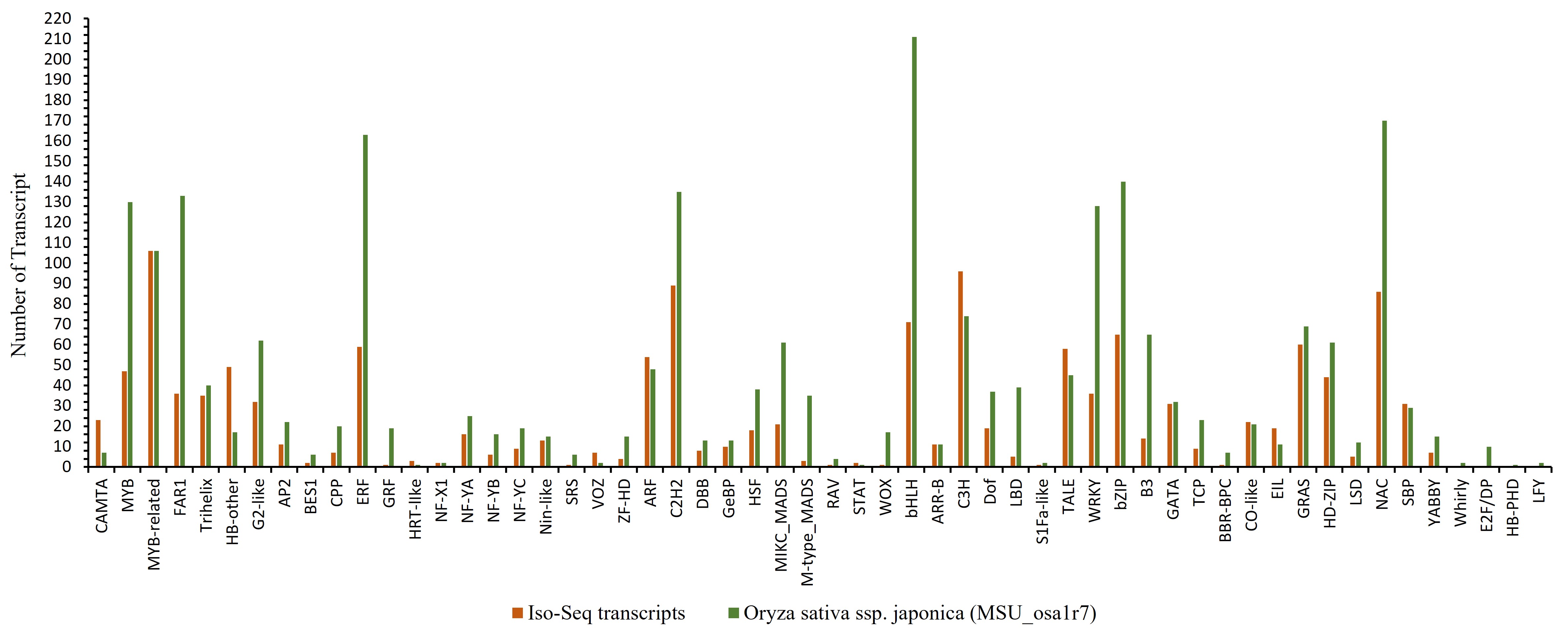

Supplement: Supplementary file 4 — Additional file 4. Fig S2: Comparison of plant transcription factors of O. sativa ssp. japonica var. Nipponbare obtained from Iso-Seq transcripts from PacBio Sequel platform and MSU Rice Genome Annotation Project Release 7, MSU (v.7) obtained from the plant transcription factor database (PlantTFDB version 5.0) (Jin et al. 2017). [file 12284_2022_577_MOESM4_ESM.jpg]

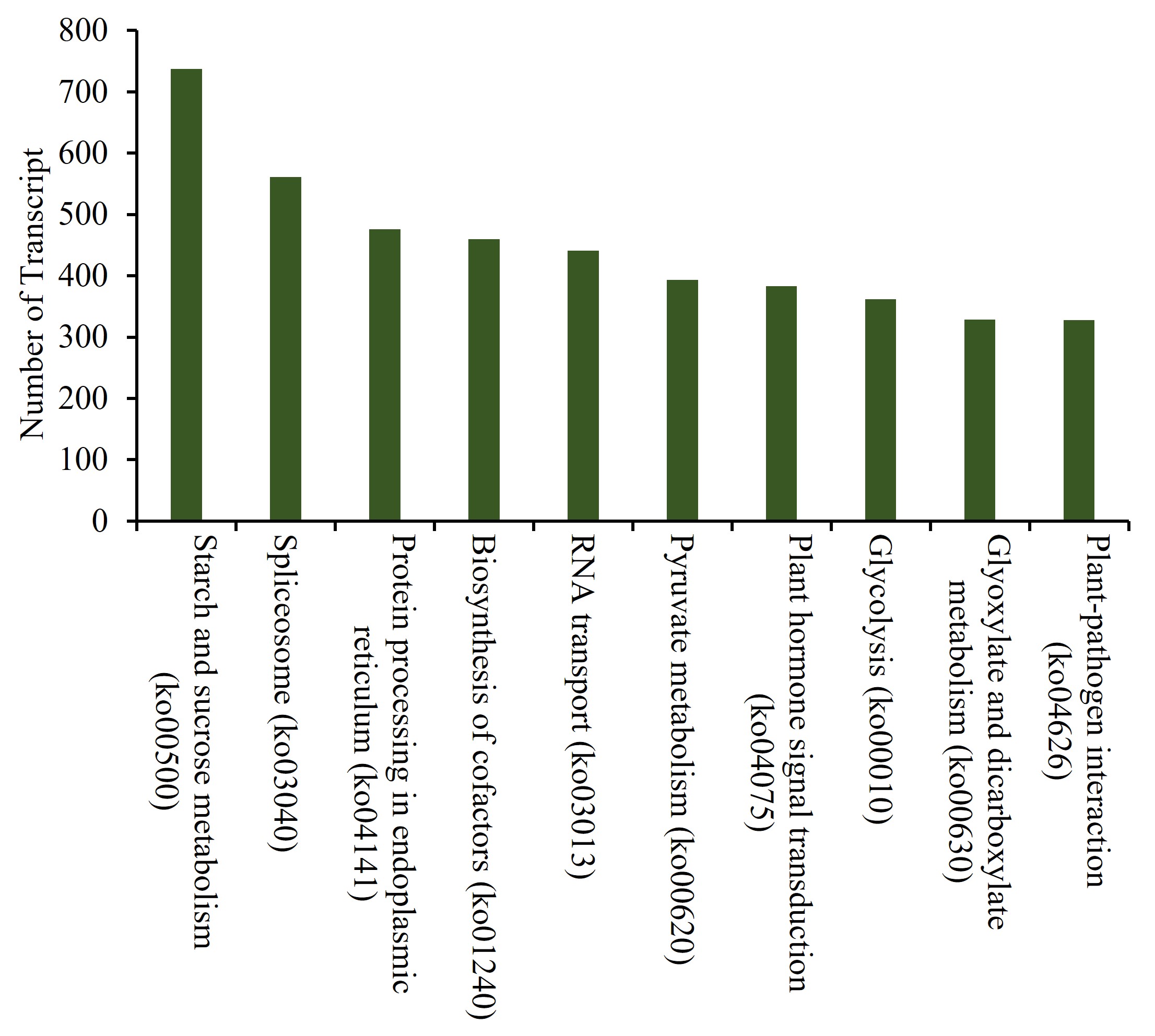

Supplement: Supplementary file 5 — Additional file 5. Fig S3: Top ten Kyoto Encyclopedia of Genes and Genomics (KEGG) pathways represented by the highest number of transcripts linked to the pathway categories of genetic information processing, cellular process, environmental information processing, organismal systems, and metabolism in all the aligned transcript isoforms. [file 12284_2022_577_MOESM5_ESM.jpg]
